# Organelle contact sites in cancer cells

**DOI:** 10.1038/s41419-026-08674-5

**Published:** 2026-04-04

**Authors:** Ilaria Celotti, Matteo Scavezzon, Sabrina Toffanin, Alessia Ruzza, Federica Vianello, Elisabetta Zaltron, Carol Bastianello, Michela Rossini, Filippo Severin, Luigi Leanza

**Affiliations:** Department of Biology, University of Padova, Padova, Italy

**Keywords:** Organelles, Cancer

## Abstract

Membrane contact sites (MCSs) are defined as regions of functional proximity between membranes belonging to the same or different organelle types. These interactions are mediated by specialised proteins promoting the formation of these crosstalk hubs. Previously, organelles were considered to act independently in cellular physiology. However, it is now evident they carry out specific functions at MCSs. The first interactions described involved endoplasmic reticulum and mitochondria. Subsequently, many contacts involving different organelles emerged. MCSs affect several cellular processes, including intracellular signalling, lipid and ion homeostasis, transport of molecules, cellular metabolism, and redox balance. Disruption of these interactions has been described to be associated with various pathologies, including cancer. While the role of MCSs in tumours remains unclear, recent findings suggest they may influence cancer progression, so, in the near future, modulating organelle interactions could provide novel therapeutic options and develop new protocol to treat tumours.

**Figure:**
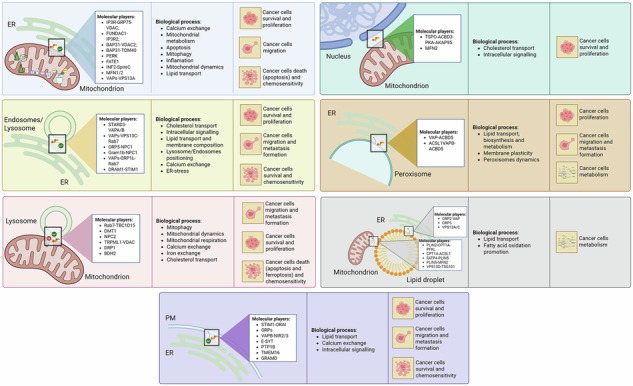
Schematic overview of intracellular MCSs, their effects on biological processes and the associated cancer-related outcomes. MCSs involve different cellular organelles allowing their intercommunication, finally participating in a plethora of cellular processes ranking from calcium and ions exchange, lipid transport and regulation of cell survival. Thus, MCSs modulation has been demonstrated to play a pivotal role in the modulation of cancer aggressiveness.

Schematic overview of intracellular MCSs, their effects on biological processes and the associated cancer-related outcomes. MCSs involve different cellular organelles allowing their intercommunication, finally participating in a plethora of cellular processes ranking from calcium and ions exchange, lipid transport and regulation of cell survival. Thus, MCSs modulation has been demonstrated to play a pivotal role in the modulation of cancer aggressiveness.

## FACTS

- Membrane contact sites (MCSs), regions of proximity between organelles, regulate key cellular processes, including calcium signalling, lipid transfer, metabolism and redox homeostasis.
- MCSs are stabilised by specialised tethering proteins that enable inter-organelle communication without membrane fusion.
- Increasing evidence links alterations in organelle contact sites to cancer cell metabolism, survival and stress responses.

## OPEN QUESTIONS

- How are organelle contact sites dynamically regulated during cancer development and progression?
- Can modulation of organelle contact sites be exploited as a therapeutic strategy in cancer?
- Which experimental approaches will allow the accurate visualisation and quantification of MCS dynamics in living cancer cells?

## Introduction

Membrane contact sites (MCSs) are defined as regions of close proximity between membranes of distinct cellular organelles. Interestingly, MCSs can involve two or three different types of organelles. Nowadays, it is clear that the composition of these MCSs is not static; rather, they are dynamic structures that undergo modifications in response to cellular stressors and metabolic demands [1–3]. All these contacts facilitate the exchange of key molecules and substrates, which impact on numerous signalling pathways and processes essential for cellular homeostasis, including calcium (Ca^2+^) signalling, lipid synthesis and metabolism, nutrient balance, autophagy, protein metabolism, stress responses and organelle biogenesis, dynamic and localization. Given their importance in such several vital processes, alterations in MCSs have been associated with several pathologies, including cancer. Specifically, MCSs modulation associates with the control of different cancer cell hallmarks, including tumourigenic uncontrolled cell growth, metabolic reprogramming and resistance to cell death, finally impacting on resistance to therapeutic agents and ability to metastatize [4]. This suggests that targeting and modulating the components of MCSs may represent a valid strategy to positively impact on tumourigenesis. Given contact site role in different biological and pathological processes, novel approaches have been developed to study and quantify contacts between organelles [5]_._

In the present review, we will discuss the structure of the up to date described inter-organelle MCSs, focusing on their impact on cancer development and progression, to highlight their role as possible oncological targets.

## ER-Mitochondria contact sites

**Fig. 1:**
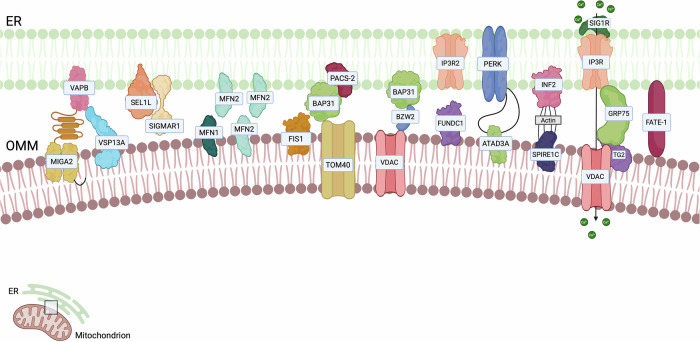
Endoplasmic reticulum—mitochondria. The outer mitochondrial membrane (OMM) hosts multiple tethers connecting it to the endoplasmic reticulum (ER). In this context, Ca^2+^ efflux and lipid transfer can modulate ER-mitochondria contact sites. Calcium signalling involves dynamic processes linking temporal fluctuations in intracellular calcium concentrations within various cellular compartments, regulated by calcium-dependent sensors and effectors.

The Mitochondria-Endoplasmic Reticulum (ER) Contact sites (MERCs), also referred to as mitochondria-associated membranes (MAMs) were the first to be observed in the 1950s [6, 7]. From 1990, they gained increasing scientific consideration, both from the structural and functional point of view [8]. MERCs represent hubs for signalling that control multiple aspects of mitochondrial biology including cellular survival [9], redox-signalling [10], Ca^2+^ release [11], lipid transfer [12], immune responses [13], inflammation [14], autophagy [15], mitochondrial dynamics [16], metabolism [17], cell death sensitivity and metastasis development [18], which all contribute to tumourigenesis [9, 19]. Considering MERCs are involved in a plethora of cellular key processes, it is not surprising that altered MERCs can play an important role also in different pathological conditions [20, 21]. Several ER-mitochondria tethers have been described so far, and they are depicted in Fig. 1. In the next paragraphs, we will discuss which of them have been shown to have an impact on cancer (Table 1).

**Table 1:** Main ER-mitochondria contact sites and their associated features.

Cargo	Main tether (ER-mito)	Biological functions	Tumour phenotype	Ref.
**Ca**^**2+**^	GRP75-IP3R-VDAC	Regulation of calcium transfer between the ER and mitochondria	Impaired Ca^2+^ transport, increased proliferation and migration	[22, 32, 33]
**Ca**^**2+**^	FUNDC1 – IP3R2	Mitophagy, Ca^2+^ transfer to mitochondria	Promotion of neo-angiogenesis, inhibition of cancer cell proliferation, apoptosis induction, increased drug sensitivity	[49, 50, 54]
**Lipids, Ca**^**2+**^	VAPs – VPS13 VAPs – MIGA2	Lipid transfer, Ca^2+^ homeostasis, autophagy, unfolded protein response (UPR), microtubule organisation, and neurotransmitter release	Increase of tumour growth	[56, 68]
	Mitofusins	Mitochondrial fusion, mitochondrial function regulation, ER homeostasis and cellular metabolism	Apoptosis induction, promotion or reduction of cancer cell invasion depending on cancer type, increased invasiveness	[70, 79, 314, 315]
**Ca**^**2+**^	BZW2	Regulation of c-Myc, ERK/MAPK, Wnt/β-catenin and Akt/mTOR pathways, Ca^2+^ fluxes, control and sorting of proteins directed to the ER	Glycolysis enhancement, impact on cancer cell metabolism, ATP production modulation, worse prognosis	[82–88]
	BAP31	Apoptosis induction, ROS production	Inhibition of cell proliferation, apoptosis induction and reduced invasiveness	[90, 91]
**Cholesterol**	PERK	UPR, modulation of ER stress, mitochondrial homeostasis	Cancer cell adaptation, increased cell survival, angiogenesis, migration, and dissemination, inhibition of immune activation	[99, 103–105]
**Ca**^**2+**^	FATE1	Mitochondrial morphology modulation and functionality, Ca^2+^ fluxes modulation	Impairment of Ca^2+^ fluxes, steroid hormone production modulation, MERCs formation, impact on cancer cell survival, cancer cell resistance to drugs	[18, 112]
	SigmaR1	Chaperone activity, ERAD pathway, involvement in VEGF-induced autocrine positive feedback mechanism	Enhancement of hERG activity, increased cell proliferation, angiogenesis promotion	[94, 95, 114, 115]
	SEL1L	ERAD pathway, modulation of PTEN transcription	Increased or decreased tumour growth and aggressiveness depending on the cancer type and metastasis formation	[116, 316, 317]
**Ca**^**2+**^	INF2	Mitochondrial constriction, actin polymerisation, Ca^2+^ transfer between ER and mitochondria.	Increased invasiveness	[121, 123, 124]

### GRP75-IP3R-VDAC

One of the most critical axes regulating Ca^2+^ mobilisation from the ER to mitochondria involves the inositol 1,4,5-trisphosphate receptor (IP3R) on the ER membrane, the Glucose-Regulated Protein 75 (GRP75 or HSPA9) [22], present in the MAMs and acting as a bridge molecule [23], and the Voltage-Dependent Anion Channel (VDAC) on the outer mitochondrial membrane (OMM). This complex is further stabilised by accessorial proteins, such as Transglutaminase type 2 (TG2) [24] and Breast Cancer Type 1 susceptibility protein (BRCA1) [25], and it can be influenced by ROS production and IP3R post-translational modifications [5]. Cancer cells can remodel Ca^2+^ exchange to favour tumourigenesis [26]. Indeed, mitochondrial Ca^2+^ uptake stimulates the tricarboxylic acid (TCA) cycle [27–29], and supports reductive carboxylation, a metabolic pathway that requires the Ca^2+^-sensitive enzyme α-ketoglutarate dehydrogenase [30], thereby promoting cancer cell survival, proliferation and migration [31–33] especially when the electron transport chain is impaired [34], a common feature in cancer cells [35, 36]. However, excessive mitochondrial Ca^2+^ accumulation triggers permeability transition pore opening, ultimately leading to apoptosis [37, 38]. These observations underscore the necessity for a fine regulation of mitochondrial Ca^2+^ signalling in determining cancer cell fate.

Thus, activation of this axis is exploited by specific tumour-suppressor genes, such as Phosphatase and Tensin Homologue (PTEN), which negatively regulates the oncogene Protein Kinase B (PKB), also known as Akt, which phosphorylates IP3R [39] and inhibits Ca^2+^ -mediated apoptosis [40, 41]. Regarding apoptosis regulation, another key factor is the promyelocytic leukaemia (PML) tumour suppressor protein, which induces cell death by interfering with Akt-dependent phosphorylation of IP3Rs, consequently enhancing the IP3R-mediated Ca²⁺ release from the ER [42]. Similarly, B-cell lymphoma 2 (Bcl-2) knock-down in ovarian cancer promotes apoptosis by implementing ER-mitochondria Ca^2+^ exchange and inducing mitochondrial Ca^2+^ overload [43].

On the other hand, in certain cancers, mutations in PTEN result in the loss of its function, which impairs Ca^2+^ transport and confers resistance to apoptosis [44]. Moreover, under ER stress conditions, the Sigma-1 receptor (SigmaR1), which is often upregulated in cancer cells, binds to IP3R, promoting the influx of Ca^2+^ inside the mitochondria [45], and the activation of Ca^2+^-dependent enzymes of the glycolysis and the TCA cycle [46].

### FUNDC1

FUN14 domain-containing 1 (FUNDC1) is an OMM member of the mitophagy receptor family [47]. Under hypoxic conditions, it accumulates at the MAMs and it interacts with calnexin, therefore promoting mitochondrial fission by facilitating the recruitment of Dynamin-1-like protein (DRP1) [48]. FUNDC1, which is often dysregulated in cancer, interacts with various proteins, including HSP70 and mitochondrial fission 1 protein (FIS1), thereby triggering mitophagy which is essential for clearing unfolded proteins. In addition, the interaction of FUNDC1 with IP3R2 plays a crucial role in the transport of Ca^2+^ to the mitochondria [49]. As a mitophagy effector, FUNDC1 contributes to cancer biology by modulating mitochondrial bioenergetics and redox homoeostasis, as well as inter-organelle communication, thereby influencing tumour progression and metastatic potential. In vivo data suggest a pro-tumourigenic role for FUNDC1 in cervical cancer, as its loss resulted in the inhibition of cellular proliferation; similarly, it has been demonstrated that FUNDC1 promotes apoptosis induced by cisplatin and ionising radiations [50]. Another in vitro study identifies FUNDC1 as an oncogenic protein involved in cholangiocarcinoma (CCA) progression. Specifically, FUNDC1 inhibition leads to mitochondrial dysfunction and promotes ferroptosis through a RAC1-dependent mechanism. Moreover, in triple-negative breast cancer, ferroptosis was recently found to be regulated by MERCs also through the modulation of local phospholipid peroxidation [51]. Disruption of FUNDC1-RAC1 interaction inhibits tumour growth in both human CCA cell lines and tumour specimens [52] and the pro-tumourigenic role for FUNDC1 is further supported by in vitro studies in breast cancer, showing that FUNDC1 is overexpressed and associated with poor prognosis; in this context, FUNDC1 positively regulates cancer cell proliferation, migration and invasion in a Ca^2+^-dependent manner [53]. Furthermore, FUNDC1 may influence cancer progression by promoting neo-angiogenesis, which is critical for cancer dissemination and metastasis formation. Indeed, a decrease in FUNDC1-dependent MAMs formation exerts negative effects on the angiogenic process, potentially due to disrupted Ca^2+^ homoeostasis and a reduced expression of the vascular endothelial growth factor receptor 2 (VEGFR2) in endothelial cells [54]. The analysis of *FUNDC1* mRNA expression levels revealed that its expression was higher in cancer groups, including breast, cervical, colorectal, lung, ovarian, pancreatic, and prostate cancers as well as leukaemia and lymphoma, respect to control groups [55]. Indeed, recent findings revealed that the ablation of FUNDC1 results in the accumulation of dysfunctional and fragmented mitochondria, consequently triggering the activation of the inflammasome and potentially attenuating hepatocarcinogenesis in mice [53].

### VAPs

Vesicle-associated membrane-protein-associated proteins (VAPs) are ER proteins that play a crucial role in inter-organelle communication [56]. VAPs can interact with proteins containing a FFAT (Two Phenylalanines in an Acidic Tract) motif, such as members of the oxysterol-binding protein (OSBP) family or phosphatidylinositol transfer proteins (PITP) from the PITPNM family [57]. Through these interactions, VAPs contribute to a range of cellular processes including organelle membrane tethering, lipid transfer, Ca^2+^ homeostasis, autophagy, unfolded protein response (UPR), microtubule organisation, and neurotransmitter release [56, 58]. Specifically in cancer, VAPs were reported to promote cell proliferation [59–61], thus potentially becoming new biomarkers and therapeutic targets [62].

VAPs sustain MERC formation by interacting with several proteins, like VPS13 (vacuolar protein sorting 13) family which is involved in lipid transport at MCSs. These proteins can bind glycerolipids and facilitate their transfer between organelles, thereby forming a direct channel between adjacent membranes [63]. Different VPS13 proteins are localised to distinct MCSs. VPS13A works as a tethering factor whose overexpression increases MERC number while its loss impairs lipid transfer between the ER and mitochondria, leading to disruptions in mitochondrial fission, fusion, and mitophagy [64]. VPS13A also plays a role in cancer since in silico data show that it is highly expressed in tumours including rhabdomyosarcoma, gastric cancer, and ovarian cancer [65].

Mitoguardin 2 (MIGA2) is a OMM protein involved in de novo triacylglycerol (TAG) synthesis and lipid transport at the MERCs by interacting with VAPA/B [66, 67]. In vivo experiments show that MIGA2 may support cellular proliferation in ovarian cancer *via* Yes-associated protein 1 (YAP1), which is regulated through the Hippo signalling pathway. Additionally, MIGA2 and YAP1 can regulate AKT activity, thereby affecting cell growth [68].

### Mitofusins

Mitofusin 1 and 2 (MFN1/2) are homologous proteins belonging to the family of mitochondrial transmembrane GTPases. Despite their similarity, they are not functionally redundant. MFN1 exhibits greater GTPase activity and stronger mitochondrial tethering capacity, whereas MFN2 has additional cellular functions, including the capacity to mediate the tethering between the ER and mitochondria [69]. Mitofusins play a fundamental role in mitochondrial fusion and in the regulation of other mitochondrial functions, thereby ensuring proper organelle morphology, functionality, and distribution within the cell [70]. Additionally, they contribute to several cellular processes, like ER homeostasis, and cellular metabolism [71].

MFN2 has been observed on the ER membrane, particularly at MERCs, where it requires mitochondrial MFN2 or MFN1 to bridge the two organelles and maintain them at an optimal distance [72, 73]. Cellular processes regulated by MFN2 include Ca²⁺ transfer [73] and ER-stress response [74]. However, the role of MFN2 at MERCs is debated, since both a tethering and a spacer function have been proposed for this protein. Specifically, MFN2 downregulation has been associated with an increased formation of short-distance MERCs and enhanced Ca^2+^ flux between the two organelles [75–78], finally impacting on different diseases, such as Alzheimer’s disease [77]. MFN2 plays a complex and context-dependent role in cancer, functioning as either a tumour suppressor or an oncogene depending on the tissue type. In several cancers, including liver, colorectal, and lung tumours, MFN2 has been shown to promote apoptosis and limit cancer cell proliferation, supporting its role as a tumour suppressor. Mechanistically, MFN2 can negatively regulate tumour growth by modulating the Ras–nuclear factor kappa-light-chain-enhancer of activated B cells (NF-κB) signalling pathway, as its deletion enhances cancer cell proliferation and invasiveness. Likewise, in vitro studies in breast cancer cells demonstrated that MFN2 suppresses proliferation by inhibiting mTORC2/Akt signalling [79]. However, MFN2 can also function as a tumour promoter. Indeed in vivo, it has been linked to increased cancer cell invasiveness, and its upregulation correlates with poorer overall survival in gastric cancer and lung adenocarcinoma [80]. These contrasting findings highlight the dual and highly context-dependent nature of MFN2 in tumourigenesis.

### BZW2 and BAP31

Basic Leucine Zipper and W2 Containing Protein 2 (BZW2) is a highly conserved protein of the basic-region leucine zipper (bZIP) superfamily of transcription factors [81]. Among the pathways influenced by BZW2 there are c-Myc, Wnt/β-catenin [82] ERK/MAPK [83] and Akt/mTOR [84]. BZW2 has been described as a new oncogene promoting the progression of different cancers. Its high expression has been documented across different tumours and it is associated with poor prognosis [85–88]. More in detail, BZW2 enhances glycolysis in lung adenocarcinoma [85]. In addition, BZW2 can interact with proteins involved in MERC formation. In pancreatic adenocarcinoma (PDAC), it can bind the ER B-cell receptor-associated protein 31 (BAP31) and VDAC2, sustaining Ca^2+^ flux and impacting on cell metabolism [85]. Since many mitochondrial metabolic enzymes are sensitive to Ca^2+^ levels, targeting this axis can guarantee a reduction in ATP levels, impairing cancer progression. In addition, a decreased TCA cycle activity has been reported upon BZW2 downregulation which is counteracted by an increase in fatty acid oxidation (FAO) rate promoted by the activation of AMPK pathway [86]. BAP31 is involved in the control and sorting of proteins directed to the ER [88]. Its cytoplasmatic domain can interact with cytoskeletal elements but also with proteins expressed on the surface of other organelles [89]. In HeLa cells, BAP31 can bind Fis1: during the first step of Fis1-induced apoptosis, BAP31 undergoes a caspase-like cleavage resulting in p20BAP31 formation. While Fis1-BAP31 tether occurs in physiological condition, Fis1-p20BAP31 interaction is necessary for the recruitment and activation of pro-caspase 8 only upon apoptosis induction. Moreover, p20BAP31 causes the release of Ca^2+^ from the ER, leading to an increase in cytosolic Ca^2+^, which is required for triggering apoptosis [90]. In colorectal cancer cells, p20BAP31 overexpression leads to an inhibition of cell proliferation, a reduction of the clonogenic potential, and an increase in apoptosis. Moreover, it is responsible for a decrease in mitochondrial membrane potential and an increase in mitochondrial ROS, finally leading to the ROS-JNK pathway and caspase activation. p20BAP31 can induce both caspase dependent and independent apoptosis since it can prompt the translocation of apoptosis-inducing factor (AIF) from mitochondria to the nucleus, finally promoting DNA fragmentation and cell death [91].

For what concerns the regulation of MAMs, Phosphofurin acidic cluster sorting protein 2 (PACS-2), a multifaceted regulator of membrane trafficking, significantly contributes to apoptosis, autophagy, and regulation of MAMs [92], acting in conjunction with Fis1 and BAP31. PACS-2 depletion is associated with mitochondrial fragmentation by stabilising p20BAP31, and is implicated in lipid synthesis and in the formation of autophagosomes. In tumour cells, PACS-2 expression is reduced or completely absent. Despite this, in colorectal cancer, PACS2 may have an indirect role in activating the epidermal growth factor receptor (EGFR), thereby promoting interleukin-6 (IL-6) production and contributing to tumour development [93].

Translocase of Outer Mitochondrial Membrane 40 (TOMM40), a protein involved in the import of proteins targeted to mitochondria, is one of the proteins interacting with BAP31 [94, 95]. In nasopharyngeal carcinoma, in vitro experiments show that TOMM40 upregulation correlates with poor prognosis, and it promotes cell proliferation through the activation of AKT/mTOR and p53/p21 signalling pathways [96]. In oral squamous cell carcinoma, TOMM40 has been proposed as a new oncogene with prognostic value since its expression is enhanced in tumours compared to normal tissues. Furthermore, it correlates with lower disease-free and overall survival [97]. In mice, the interaction between TOMM40 and BAP31 has been documented and the loss of BAP31 seems to promote autophagy, mitophagy and mitochondrial fragmentation and dysfunction, since it takes part in Complex I assembly [98].

### PERK

Protein Kinase RNA-Like ER Kinase (PERK) is mostly located at MERCs and plays a role in the UPR in cooperation with IRE1 and ATF6 [99]. On one hand, PERK loss appears to confer a protective mechanism against apoptosis mediated by ROS-induced ER stress by disruption of MERCs [100] while increasing accumulation of F-actin, which may impair the formation of contacts between the ER and the plasma membrane (PM) [101]. Additionally, PERK expression increased phosphorylated eIF2α (p-eIF2α) and promoted G0–G1 arrest and cancer cell survival in vitro, negatively impacting on tumorigenesis [102]. On the other hand, the contacts established through the tethering complex PERK–Ero1a enhance communication between the ER and mitochondria, promoting Ca²⁺ flux that supports the enzymatic activity of TCA cycle and OXPHOS enzymes, ultimately increasing cell viability. PERK activity is further regulated by MFN2, another tethering protein that interacts with PERK to establish ER–mitochondria contacts. Moreover, PERK enables cancer cells to adapt to adverse conditions within the tumour microenvironment. By facilitating the translation of activating transcription factor 4 (ATF4), the PERK-ATF4 axis promotes cell survival, angiogenesis, migration, and dissemination of cancer cells [103]. In human breast cancer tissues, PERK is often constitutively activated through phosphorylation, and this activation is associated with an increased risk of distant metastasis development [104]. It has also been proposed that PERK activation exerts an inhibitory effect on immune activation in certain cancer types. In melanoma cells, PERK inhibition impairs the ability to manage ER stress, resulting in paraptosis-mediated immunogenic cell death, which facilitates the differentiation of monocyte precursors [105]. In triple-negative breast cancer cells, reduced proliferation has been shown to correlate with PERK loss, potentially due to ROS accumulation and oxidative DNA damage, or to inactivation of Nrf2 signalling [106].

Upon activation, PERK can also interact with ATPase Family AAA Domain Containing 3A (ATAD3A), a protein involved in mitochondrial homeostasis and ER–mitochondria signal transduction [107], as well as in ER stress modulation, cholesterol trafficking, and cancer metastasis [108]. The binding of ATAD3A to PERK results in the attenuation of PERK signalling, due to a reduced binding affinity for eIF2α [109]. Upregulation of both PERK and ATAD3A correlates with an increase in MAM formation, pointing out their involvement in the establishment of MERCs [109]. In cancer, ATAD3A is overexpressed in various malignancies, including hepatocellular carcinoma (HCC), head and neck squamous cell carcinoma (HNSCC) and lung adenocarcinoma, and it is associated with poor prognosis and decreased survival rates. Conversely, in breast cancer, ATAD3A overexpression determines the inhibition of cell growth and lower levels of ATAD3A expression correlate with reduced patient survival, supporting its tumour-suppressor function. This variability in the impact of ATAD3A expression suggests that its role may differ among distinct tumour types [110].

Similarly, by interacting with multiple protein partners and acting as a UPR sensor, PERK may differently modulate oncogenic processes depending on the molecular context, such as the prognostic relevance of PERK that varies across tumour types. High PERK expression is associated with poor prognosis in KIRP, LGG, BRCA, and THCA, but with favorable prognosis in HNSC. GSEA results indicate that PERK is mainly enriched in immune-related signalling pathways in BRCA, HNSC, and THCA. PERK expression positively correlates with macrophage infiltration, suggesting that elevated PERK levels may promote immune cell infiltration into the tumour microenvironment and serve as a potential prognostic marker in some cancers. Consistently, another report showed that the inhibition of the PERK–eIF2α–GRP94 signalling pathway suppresses EGFR expression and enhances radiosensitivity of Oral Squamous Cell Carcinoma (OSCC) cells [111].

### Other ER-mitochondria tethers

Foetal and Adult Testis-Expressed 1 (FATE1) is a protein predominantly localised in the testis within normal tissues, although it has been identified in various cancer types. FATE1 is situated at the MERCs, anchored to the ER, and it interacts with Emerin and Mitofilin on OMM. Thanks to this interaction, it influences mitochondrial morphology and functionality [18]. FATE1 has been shown to increase MERC distance or to disrupt them. Indeed, FATE1 appears to adversely affect Ca^2+^ fluxes, and steroid hormone production in adrenocortical carcinoma (ACC) thereby enhancing the resistance of cancer cells to apoptosis induced by chemotherapeutic agents [18, 112, 113].

The Sigma 1 receptor (SigmaR1) is a chaperone protein located on the MAMs, nuclear membrane, and plasma membrane. SigmaR1 serves as a substrate within the ER-Associated Degradation (ERAD) pathway, which is regulated by SEL1L (Suppressor/Enhancer of Lin-12-Like), and it is crucial for maintaining ER protein quality control. SigmaR1 engages with various ion channels across different families, thereby playing a significant role in the regulation of intracellular Ca^2+^ fluxes and in the formation of MERCs [94, 114]. SigmaR1 is overexpressed in chronic myeloid leukaemia (CML), acute myeloid leukaemia (AML) and colorectal cancer, where tumour progression correlates with hERG expression. SigmaR1 directly interacts with hERG, thereby enhancing its activity [95]. Additionally, a positive role for SigmaR1 in facilitating angiogenic processes has been proposed, as it is involved in a VEGF-induced autocrine positive feedback mechanism [115]. Similarly, in tumour progression, SEL1L regulates the ERAD pathway to maintain protein homeostasis. In response to ER stress, this pathway is activated to degrade unfolded proteins through the proteasome, finally restoring ER homeostasis. Indeed, both in vitro and in vivo studies indicate that SEL1L prevents the accumulation of misfolded or aberrant proteins that would otherwise promote tumour aggressiveness [116]. SEL1L is a multifunctional regulator whose impact on tumour biology is highly context- and cancer-dependent. While SEL1L is widely expressed in normal tissues, its expression is frequently reduced or lost in several tumour types, suggesting a context-dependent role as either an oncogene or a tumour suppressor. In pancreatic cancer cells, for example, loss of SEL1L reduces tumour growth and aggressiveness, an effect attributed in part to its modulation of PTEN transcription and subsequent influence on early metastatic processes [81]. SEL1L can also function as a negative regulator of Notch signalling, further implicating it in key pathways governing tumour progression [82]. Consistent with these observations, SEL1L expression in breast and pancreatic carcinomas has been associated with decreased cellular aggressiveness. In breast cancer, SEL1L appears to inhibit Epithelial-Mesenchymal Transition (EMT) by promoting Sonic hedgehog (Shh) degradation, whereas in pancreatic cancer its tumour-attenuating function has been linked to the regulation of the TGF-β pathway [117, 118]. In contrast, SEL1L overexpression correlates with favourable clinical features in certain malignancies. In colorectal cancer, increased SEL1L levels have been associated with well-differentiated tumour cells and decreased aggressiveness, indicating that SEL1L upregulation is predictive of a better prognosis [119]. However, the relationship between SEL1L expression and tumour behaviour differs across cancers. In several gliomas, SEL1L upregulation has been reported in association with TERT promoter mutations and EGFR amplification, molecular alterations that are considered negative prognostic markers in these tumours [120].

Inverted formin-2 (INF2) is an ER protein involved in mitochondrial constriction and it can assemble actin subunits into filaments, to sustain the contraction process [121]. INF2 interacts with Protein spire homologue 1 (Spire1C) during the formation of actin filaments [122]. Additionally, by promoting MERCs, INF2-mediated actin polymerisation is essential for Ca^2+^ transfer between ER and mitochondria [123]. Importantly in cancer, it is well-established that actin structures significantly contribute to the invasive properties of tumour cells [124]. Experiments on MDA-MB-231 cells have demonstrated that an increase in Spire1 enhances cancer cell invasion of the endothelial monolayer, indicating that this protein, along with other ones involved in actin nucleation, plays a role in promoting cancer invasiveness [125].

## ER-lysosome contact sites

Despite the close relationship with mitochondria, the ER is involved in other contacts with different organelles, such as endosomes and lysosomes [126]. Endocytosis is a cellular process through which cells internalise extracellular material or PM components through the formation of intracellular vesicles called endosomes. Endosomes are classified based on their grade of maturation in early and late endosomes [127]. While moving toward lysosomes, endosomes undergo a maturation process involving a progressive pH acidification mediated by Golgi-derived proton pumps. While early endosomes show Rab5 as one of their main membrane proteins, upon endosome maturation, Rab5 is displaced by Rab7 which is involved in the interaction between endosomes–lysosomes and ER. Moreover, the lipid PI(3)P of early endosomes is phosphorylated to PI(3,5)P in late endosomes, thus affecting the recruitment of sorting and tethering factors [128]. The existence of tight and stable connections between ER and endosomes has been investigated through electron microscopy [129]. Endosomes associate with the ER early during their maturation process and the number and duration of these interactions increase as endosomes mature. Indeed, in Cos-7 cells ER rearranges its structure to maintain this interaction through the opening or closure of membranous rings, usually at the three-way junction [130]. Importantly, the contacts with the ER control the position of endosomes within the cell [129]. Endosomes mainly localise in the perinuclear region and display a static behaviour. However, a small number of intracellular vesicles are highly dynamic and move towards the periphery of the cell. E3 ubiquitin Ligase Ring finger protein 26 (RNF26) is a protein localised on the ER proximal to the nucleus and it is involved in the late endosomes’ spatial organisation and vesicles maturation in the perinuclear region in vitro [131].

**Fig. 2:**
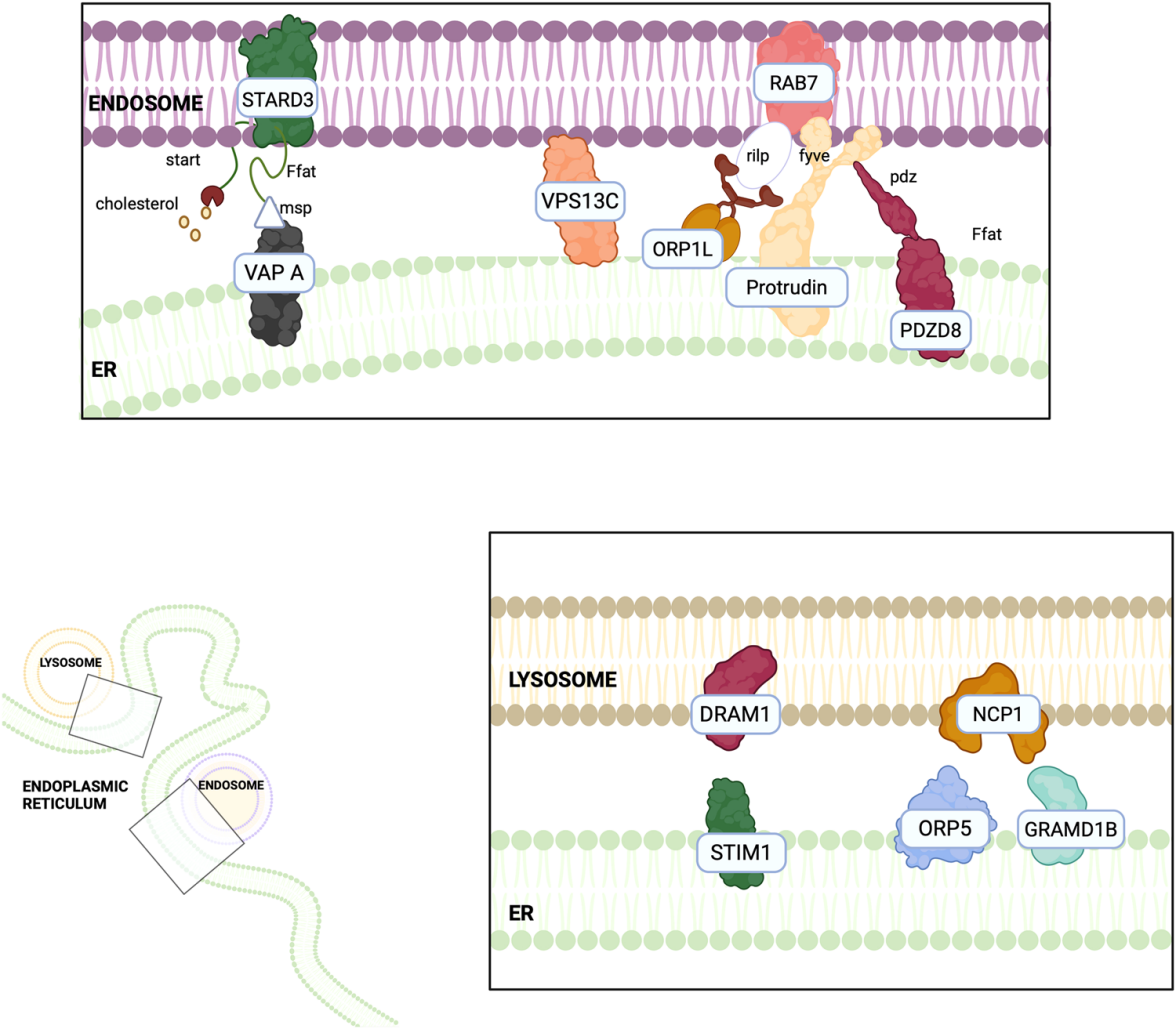
Endoplasmic reticulum-Lysosome. Numerous tethers exist between the ER and various intracellular organelles, including endosomes and lysosomes. These interactions are important since the association with the ER influences endosome localisation. ER–late endosome contact sites, involving STARD3 and VAP proteins, play a key role in cholesterol trafficking and impact on cancer progression by altering lipid metabolism. Other proteins such as ORP5, NPC1, and Protrudin influence endosome movement and the release of enzymes that promote tumour invasion.

Interestingly, an increasing number of studies are demonstrating the biochemical heterogeneity of ER-endosome/lysosomes contacts, highlighting the presence of different tethering axes both in different cellular types and in the same cellular contexts (Fig. 2). Hence, the ER-endosomes associations have been proven to participate in different cellular processes ranging from endosomal positioning [131] to endosomes fission events [132], lipid and cholesterol transport [133], ion homeostasis [134] and intracellular signalling [135]. In the next paragraph, we will discuss the tethers involved in ER-endosome/lysosomes contacts in cancer (Table 2).

**Table 2:** Main ER-lysosomes contact sites and their associated features.

Cargo	Main tether (ER-lyso)	Biological functions	Tumour phenotype	Ref.
**Cholesterol**	STARD3-VAPs	Scaffold ER-endosome contacts, delivery of cholesterol to endosomes	Alterations in cholesterol metabolism, modulation of membrane permeability and fluidity, transduction of survival signals, increased proliferation, metastasis formation, blockade of lysosomal degradation, androgen biosynthesis	[137, 138, 142, 144, 318–320]
**Cholesterol**	ORP5-NPC1	Cholesterol delivery to ER, lipid metabolism modulation	Dysregulated lipid metabolism, increased cancer cell invasion and tumour progression, decreased overall survival	[148, 149, 151, 152]
**Cholesterol**	ORP1L-VAPs	Cholesterol transport to ER, regulation of endosome dynamics, formation of late endosome contact site	Modification of lysosome positioning, cancer cell autophagy, enhanced cancer cell growth, invasion and metastasis	[158–161]
	Protrudin	Promotion of cell protrusion and neurite outgrowth, kinesin-driven transport of LEs	Maturation and elongation of invadopodia, cancer cell invasion	[159, 164, 321]
**Ca**^**2+**^	DRAM1-STIM1	Regulation of autophagy, apoptosis, and cellular stress responses, the SOCE pathway	Impaired Ca^2+^ homeostasis, ER stress, increased or decreased cell proliferation, migration, invasion, depending on the tumour type, apoptosis inhibition	[167, 170, 171, 173, 322, 323]

### STARD3-VAPs and their interactors

Steroidogenic acute regulatory protein (StAR)-related lipid transfer domain containing 3 (STARD3), which is located at the membrane of late endosomes, is a scaffold protein in ER–endosome contacts, and it interacts with VAPA/B [136, 137]. In HeLa cells, elevated STARD3 expression is associated with the accumulation of sterols in the late endosomes, whereas the silencing of VAPA/B determines the inability to accumulate cholesterol in endosomes [138]. The STARD3‑VAP axis has the potential to contribute to cancer progression, as alterations in cholesterol metabolism can modulate membrane permeability and fluidity, and the transduction of survival signals, which are processes implicated in tumour development (e.g. via enhanced oncogenic signalling linked to STARD3‑mediated cholesterol redistribution in cancer cells) [139, 140]. Indeed, cancer cells exhibit elevated levels of intracellular cholesterol compared to nontumoural ones [141]. Elevated STARD3 levels are associated with cancer proliferation and metastasis formation both in vitro in breast cancer cells, and in vivo, especially in hormone-driven cancers, such as breast and prostate cancer, where it induces an independent steroidogenesis process [137]. Additionally, an overexpression of STARD3 may be associated with a blockade of the late endosome formation, and the consequent inhibition of their maturation into lysosomes thus preventing lysosomal degradation [142]. This is relevant in breast cancer, since it implies that some growth factor receptors, including HER2, are not degraded and can sustain an uncontrolled cellular growth [143]. Moreover, elevated STARD3 levels have also been detected in prostate cancer patients, where its high expression is associated with an increased transport of cholesterol to mitochondria and consequently to an enhancement of androgen biosynthesis [144].

In addition to STARD3, VAP proteins can bind to a plethora of proteins, like VPS13C, a member of the VPS13 protein family, which localises at contacts between the ER and late endosomes/lysosomes. VPS13C can interact with VAP and with the late endosomal GTPase Rab7, further supporting its role in endo-lysosomal tethering and function [145]. Mutations in the *VPS13C* gene are connected to endometrial, gastric, and colorectal cancers. Moreover, the loss of VPS13C is associated with the development of resistance to cisplatin in cervical cancer cells, suggesting a role in mediating chemotherapy sensitivity [146].

Among the VAP interactors, Oxysterol-binding protein (OSBP) and its related proteins (ORPs) are part of a large family of lipid-binding proteins which transport lipids between cellular membranes in a non-vesicular manner [147, 148]. Among them, ORP5 can bind lipids and target organelle membranes to mediate sterol signalling and transport [149]. ORP5 interacts with Niemann-Pick type C protein 1 (NPC1), localised in the late endosomes/lysosomes membrane, at ER-lysosome contact sites. NPC1 plays a crucial role in regulating contact formation between the ER and late endocytic vesicles: the knockdown of NPC1 reduces these contact sites, while NPC1 overexpression exerts the opposite effect [150]. ORP5-NPC1 interaction contributes to extract cholesterol molecules from the late endosomes/lysosomes membrane and deliver them directly to the ER in vitro [148, 151]. Both NPC1 and ORP5 are highly expressed in several cancer types. ORP5 is overexpressed in pancreatic and lung cancer cells, where it has been linked to increased cancer cell invasion and tumour progression [149]. Similarly, NPC1 is upregulated in breast cancer, and its elevated expression correlates with a decreased overall survival in glioma and an increased risk in oesophageal cancer [152]. Although the precise mechanisms by which NPC1 and ORP5 contribute to cancer progression remain unclear, it is hypothesised that their overexpression may enhance lipid transfer activity, leading to dysregulated lipid metabolism, altered membrane lipid organisation, and aberrant cell signalling [153, 154]. NPC1 can also interact and form contacts with the ER-localised sterol transport protein Gramd1b [150]. Interestingly, Gramd1b has also been implicated in different malignancies. In ovarian cancer, for example, it has been associated with chemoresistance [155], while in gastric cancer, it promotes cell survival by upregulating the expression of the anti-apoptotic protein Bcl-xL [156]. Moreover, JAK/STAT signalling positively regulates Gramd1b expression in both breast and gastric cancer cells [156, 157].

ORP1 exists in a short (S) and in a long (L) isoform and the ORP1L can interact with Rab7 regulating the positioning of the endosomes [133]. By interacting with VAPs, ORP1L functions as a late endosomal protein involved in cholesterol transport to the ER, the regulation of endosome dynamics, and the formation of late endosome (LE)-ER contact sites [158, 159]. In melanoma cells, ORP1L has been shown to sense endosomal cholesterol levels and, consequently, to modify lysosomal positioning in the cell [160]. The ability of this tether to regulate lysosomal localisation is particularly relevant in cancer, as lysosome distribution significantly influences the biological properties of cancer cells [161], since plays a crucial role in cancer cell autophagy [162], and has been associated with enhanced cancer cell growth, invasion, and metastasis [161]. Moreover, in prostate cancer cells, Rab7 is significantly downregulated, leading to lysosome redistribution toward the cell periphery, thereby supporting cancer cell invasiveness and migration [163].

Finally, Protrudin is an ER protein that promotes cellular protrusion and neurite outgrowth by mediating the plus-end trafficking of late endosomes along microtubules [159]. Thanks to its structure, Protrudin simultaneously interacts with endosomal PI3P, Rab7, and with VAPA, thereby promoting the formation of ER–endosome contact sites [129, 164]. Protrudin also harbours a binding domain for Kif5b (kinesin-1 heavy chain), which allows its recruitment to the ER–late endosome membrane contacts, supporting the kinesin-driven transport of late endosomes toward the cell periphery. Their accumulation at the plasma membrane leads to endosome fusion, thereby promoting cellular protrusions formation [165]. In cancer cells, the formation of extracellular protrusions in PM, called invadopodia, allows the secretion of matrix metalloproteinases to degrade the extracellular matrix (ECM), a crucial point for breaching the basement membrane and invading [166]. In breast cancer cells, Protrudin plays a pivotal role in the maturation and elongation of invadopodia by mediating late endosomes/lysosome translocation to the cell periphery and their subsequent fusion with the PM [164]. These findings highlight the critical role of Protrudin in cancer cell invasion. Notably, high Protrudin expression correlates with reduced survival in patients with breast, gastric, and ovarian cancers, highlighting its potential as a therapeutic target [164].

### DRAM1-STIM1

DNA Damage-Regulated Autophagy Modulator 1 (DRAM1) is a lysosomal protein identified as a regulator of autophagy, apoptosis, and cellular stress responses. As a transcriptional target of p53, DRAM1 plays a crucial role in p53-dependent autophagic and apoptotic pathways, which are fundamental in tumourigenesis [167]. Recent studies have demonstrated that DRAM1 interacts with stromal interaction molecule 1 (STIM1), a Ca^2+^ sensor located in the ER, ultimately facilitating lysosome-ER tethering. Through this interaction, DRAM1 modulates ER structure and function [168]. Moreover, STIM1 is responsible for monitoring and replenishing ER-luminal Ca²⁺ levels *via* store-operated Ca^2+^ entry (SOCE), thereby maintaining Ca^2+^ homeostasis. Loss of STIM1 disrupts this balance, leading to impaired Ca²⁺ homeostasis, to the activation of Ca^2+^ signalling, to increased ER stress, and to the subsequent induction of autophagy [169]. The DRAM1-STIM1 interaction interferes with STIM1-mediated Ca²⁺ homeostasis by reducing intracellular, mitochondrial, and ER Ca²⁺ levels while increasing lysosomal ones. This occurs through DRAM1 sequestering STIM1, therefore facilitating the transfer of Ca²⁺ from the ER to lysosomes and triggering ER stress and ER-phagy in U2OS and HEK-293T cells [168]. Interestingly, in non-small cell lung cancer (NSCLC) patients, DRAM1 is downregulated, whereas its overexpression suppresses cancer cell proliferation in vitro and in vivo, as well as cell migration, and invasion [134]. Its anti-tumour activity is associated with its function as a p53 target gene, which promotes apoptosis and autophagy activation, ultimately limiting tumour growth. Moreover, DRAM1 was reported to regulate the PI3K/Akt/mTOR pathway, exhibiting both pro- and anti-tumourigenic functions across the literature. In particular, DRAM1 was found to inhibit the PI3K/Akt/mTOR signalling pathway in NSCLC cells, likely through its enhanced autophagic flux and lysosomal enzyme activation [170]. In contrast, in gastric cancer cells DRAM1 promoted PI3K/Akt/mTOR activation and tumour progression [171], reflecting the possible context-dependent functional effects of DRAM1 on signal transduction and metabolism [172]. In glioblastoma cells, DRAM1-driven modulation of autophagy has been reported to promote tumour growth [173]. STIM1 likewise displays heterogeneous roles across malignancies. It is broadly associated with cancer progression and increased invasiveness, and in many contexts, it exerts an inhibitory effect on apoptosis [135]. STIM1 also serves as an important regulator of autophagy, with elevated intracellular Ca²⁺ promoting autophagy in hepatocellular carcinoma cells [136]. Nonetheless, contradictory findings exist, as STIM1 inhibition has been shown to induce autophagy [174].

## Mitochondria-lysosomes contact sites

**Fig. 3:**
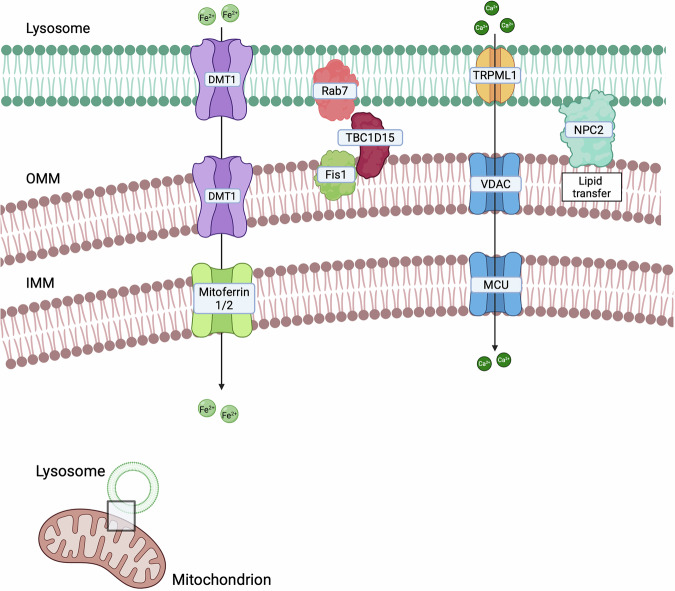
Lysosome-Mitochondria. Mitochondria-lysosomes interactions, regulated by Rab7 and TBC1D15, modulate mitochondrial behaviour and autophagy, both critical in cancer development and resistance to treatments.

Several studies have also demonstrated the formation of mitochondria-lysosomes contacts [175] (Fig. 3), mainly regulated by Rab7, which alternates between an active, lysosome-localised GTP-bound state, and an inactive, cytosolic GDP-bound state. The Rab7-GTP hydrolysis involves the recruitment of the cytosolic GTPase-activating protein (GAP) TBC1D15 to mitochondria and its interaction with the lysosomal GTP-bound Rab7, promoting its hydrolysis to the GDP-bound state. This transition prevents Rab7 from binding to its effectors, leading to its release from the lysosomal membrane and subsequent contact untethering [7]. Mitochondria-lysosome contacts are involved in mitophagy, which is often deregulated during cancer progression [172]. However, mitochondria-lysosome contact formation is not exclusively linked to mitophagy [176], but it is also crucial for regulating both organelle dynamics [175]. Lysosomal function is modulated through Rab7: following Rab7-GTP hydrolysis, the release of Rab7 effector proteins from the lysosomal membrane influences lysosomal positioning and trafficking [177]. Mitochondrial dynamic is also regulated at mitochondria-lysosome contacts, where they mark sites of mitochondrial fission and play a role in controlling the rate of fission events [175]. Furthermore, these contacts serve as platforms for the exchange of Ca^2+^, lipids, and iron between the two organelles [177]. Mitochondria-lysosomes tethering regions can also be regulated by specific signals. Indeed, under hypoxic conditions, these contacts facilitate microfusions with endolysosomes, leading to the post-translational cleavage of VDAC1 by endolysosomal enzymes. This cleavage protects mitochondria from mitophagy, thereby promoting cell survival during hypoxic stress [178]. Additionally, oxidative stress enhances mitochondria-lysosomes interactions, mediating the transfer of lipids and metabolites between the organelles to reduce apoptosis and support mitochondrial repair [179]. In the next paragraphs, we will discuss which of the already discovered tethers have been shown to have an impact on cancer (Table 3).

**Table 3:** Main mitochondria-lysosome contact sites and their associated features.

Cargo	Main tether (mito-lyso)	Biological functions	Tumour phenotype	Ref.
	LAMP1-TOM20	Lysosomal integrity protection, vesicle fusion, regulation of ATP production and maintenance of mitochondrial membrane potential	Increase in metastatic potential, cell proliferation and migration, cell cycle control and apoptosis modulation.	[324–329]
	DMT1	Iron homoeostasis (iron transfer to mitochondria)	Enhancement of invasiveness and migration, mitophagy inhibition, increase in mitochondrial respiration and glycolysis.	[180]
**Lipids**	NPC2	Lipid trafficking	Increased or decreased cancer cell proliferation and migration depending on the tumour type	[185, 189, 190, 192, 330]
**Ca**^**2+**^	TRPML1-VDAC/MCU	Ca^2+^ flux into the mitochondrial matrix	Ferroptosis inhibition, stemness rescue, regulation of oncogenic autophagy	[193, 195]
	Rab7-TBC1D15-Fis1	Mitophagy regulation	Drug sensitivity enhancement, increased cancer aggressiveness, mitophagy promotion	[197, 200, 201, 203]

### DMT1

Divalent Metal Transporter 1 (DMT1) is involved in iron homeostasis by mediating the transient interaction between endosomes and mitochondria for direct iron translocation [180]. This process is essential for limiting the intracellular labile iron pool, thereby reducing the production of ROS. Epidemiological data suggest that elevated iron levels constitute a risk factor for various cancers, including breast cancer [181]. Consistent with the central role of iron trafficking in tumour biology, DMT1 functions as a molecular bridge connecting endosomal membranes with the OMM, thereby facilitating the transfer of iron to mitochondria. This process supports mitochondrial bioenergetics and promotes breast cancer invasive cellular migration, and indeed, increased expression of DMT1 in breast cancer cells correlates with poorer overall survival [180]. Overexpression of DMT1 enhances mitochondrial iron uptake, whereas its silencing disrupts endosome–mitochondria interactions, resulting in reduced mitochondrial iron import, an elevated intracellular labile iron pool, increased mitochondrial superoxide production, induction of mitophagy, and a decline in both mitochondrial respiration and glycolysis [180]. Such regulatory effects on endosome–mitochondria communication have been observed in MDA-MB-231 triple-negative breast cancer cells, but not in the non-invasive luminal A T47D line [180]. Intriguingly, functional studies suggest that the effects of DMT1 downregulation are highly context dependent. In breast cancer models, DMT1 can increase metastatic potential; in particular, in MDA-MB-231 cells DMT1 downregulation promotes lung metastasis overgrowth in vivo, indicating that DMT1-dependent iron metabolism contributes to the malignant phenotype [180]. These findings suggest that although DMT1 supports metabolic functions associated with invasiveness, its absence may activate compensatory pathways associated with metastatic dissemination. Evidence from head and neck cancer (HNC) cell lines shows that DMT1 silencing favours ferroptosis, accompanied by iron accumulation through lysosomal sequestration, thereby triggering an iron-starvation response [182]. DMT1 is also upregulated in colorectal cancer tissue, where the resulting increase in iron flux appears to promote tumour initiation and sustain in vivo activation of the oncogenic JAK1–STAT3 signalling pathway [183]. In contrast, in HCC, DMT1 expression is associated with suppressed glycolysis and oxidative phosphorylation in silico, and correlates with better patient prognosis [184]. Collectively, these findings underscore the complex and context-dependent roles of DMT1 in cancer, highlighting its involvement in mitochondrial metabolism, iron homeostasis, cell survival, and metastatic progression.

### NPC2

NPC2 plays an important function on contacts between mitochondria and endo-lysosomes, where it mediates lipid trafficking. Indeed, NPC2 depletion in cells expressing functional NPC1 resulted in reduced mitochondrial cholesterol levels [185]. Accordingly, NPC2 ablation in HEK cells leads to an accumulation of unesterified cholesterol within late endosomes/lysosomes. Loss of NPC2 has been associated with alterations in the number of mitochondria-lysosomes contacts [186], even if this is debated [187]. NPC2 exhibits a context-dependent role in cancer, acting either as a tumour promoter or as a tumour suppressor depending on tissue type. In HCC and lung adenocarcinoma, NPC2 is downregulated, and when it is overexpressed inhibits cell proliferation and migration [188, 189]. Similarly, in glioblastoma, NPC2 overexpression inhibits tumour growth and cell migration, indicating a protective role [190, 191]. Conversely, in gastric cancer, NPC2 levels are significantly elevated in patients and in gastric cancer cells, and its absence reduces cell proliferation. In other cancers, including breast, colon, and lung cancers, NPC2 is often overexpressed and promotes proliferation, migration, and invasion [192]. These findings highlight the dual and tissue-specific role of NPC2 in tumour context.

### TRPML1-VDAC/MCU

Transient receptor potential mucolipin 1 (TRPML1) is a non-selective ion channel which is expressed on the surface of lysosomes and late endosomes that mediates Ca^2+^ efflux [193]. TRPML1 loss-of-function mutation has been associated with many neurological and lysosomal disorders, including Mucolipidosis IV (MLIV) [194]. In these pathologies, TRPML1 activity not only modulates the mobilisation of Ca^2+^, but it affects the mitochondria-lysosomes contacts too [194]. TRPML1 has been proven to be involved in different cancers like breast cancer, where it inhibits ferroptosis in cancer stem cells and reduces their stemness [193]. TRPML1 is also a key player in the regulation of oncogenic autophagy in breast and pancreatic cancer cells [195]. Its involvement in mitochondria-lysosomes contacts highlights its capability to modulate Ca^2+^ influx into the mitochondrial matrix, as shown in cervical and colon cancer cells. The modulation of mitochondria-lysosomes interaction correlates with an increase in Ca^2+^ efflux from lysosomes to mitochondria, through VDAC1 and MCU (mitochondrial Ca^2+^ uniporter), which does not cause the permeability transition pore opening neither the activation of apoptotic pathways [194]. Moreover, in TNBC cells, TRPL1 downregulation is associated with reduced mitochondria-lysosome contact sites, counteracted by increased ER-mitochondria proximity, ultimately resulting in metabolic stress, G0/G1 cell cycle arrest, cell death and enhanced chemosensitivity [196].

### Rab7-TBC1D15-Fis1

Rab7 has been described as one of the main tethers between mitochondria and lysosomes [175]. Its GTPase activity modulates mitochondria-lysosomes crosstalk, through TBC1D15 which is recruited at mitochondria by Fis1. In addition, in cervical cancer, neuroglioma, human embryonic kidney and colorectal cancer cells, mitochondria fission events occur predominantly at mitochondria-lysosomes contacts, where Drp1 is also located [175]. The role of Rab7 has been investigated in different types of cancer. For example, its downregulation in ovarian cancer leads to cisplatin resistance due to a dysregulation of the endocytic pathway that leads to cisplatin efflux [197]. Moreover, chemo-resistant ovarian cancer cells display an impaired lysosomal biogenesis and mitochondria quality control which increases the secretion of extracellular vesicles. Furthermore, in PDAC, Rab7 upregulation has been described as a poor prognostic marker [198]. Its expression has been demonstrated to be influenced by the level of NDUFS3, a subunit of mitochondrial complex I involved in its assembly. As a consequence of NDUFS3-mediated Rab7 downregulation, the endocytic pathway and mitochondrial morphology are impaired, and cancer cells acquire a less aggressive phenotype [199]. In addition, Rab7 is upregulated in gastric tumour when compared to normal tissue and it promotes cancer aggressiveness [200]. Similarly, a role for Rab7 both in vitro and in vivo in driving melanoma progression has been suggested, finally impacting on the metastatic risk [201].

The role of Rab7 in mitochondria-lysosome contacts has been well characterised in HCC where it participates in the regulation of mitophagy. In these cells a more fragmented mitochondrial network can be observed if compared to non-tumoural cells and this reflects a possible involvement of mitophagy in the progression of the disease [202]. After being phosphorylated, Drp1 is recruited to the OMM, where it promotes the division of mitochondria, which represents the starting point for mitophagy [203]. In colon cancer patient-derived cells, fatty acids promote ERK-dependent Drp1 phosphorylation enhancing mitophagy and cancer cell metabolic plasticity, finally impacting on intracellular signalling by stabilising β-catenin *via* FAO-dependent acetylation [204]. In HCC cells, Drp1 expression levels are higher than normal tissues and correlate with poorer outcomes, and Drp1 overexpression leads to its translocation at mitochondria-lysosomes contacts where it interacts with Rab7 and mediates PINK1-Parkin-dependent mitophagy [205]. In hypoxic conditions, Drp1 inhibition can enhance the sensitivity of HCC cells to apoptosis [206]. Therefore, targeting this axis can lead to HCC cells sensitivity to anti-tumoural therapies by promoting apoptosis [205].

### BDH2

Mitochondria-lysosome contact sites have also been described as platforms for iron exchange between these two intracellular compartments [207]. The enzyme 3-hydroxybutyrate dehydrogenase type 2 (BDH2) catalyzes the production of 2,5-dihydroxylbenzoic acid (2,5-DHBA), a siderophore involved in mitochondrial iron import [208]. BDH2 localises at mitochondria-lysosome contact sites and, together with its product, contributes to the regulation of intracellular iron homeostasis and trafficking. In melanoma, iron redistribution between mitochondria and lysosomes has been demonstrated to correlate with tumour phenotype [207]. Specifically, melanoma can be classified into a proliferative and differentiated phenotype characterized by an high microphthalmia-associated transcription factor (MITF), and an invasive, poorly differentiated phenotype with low MITF levels [209] which is also characterised by reduced BDH2 expression [207]. In this context, decreased BDH2 expression correlates with reduced mitochondrial iron content and the consequent accumulation in lysosomes, leading to impaired activity of mitochondrial iron-dependent enzymes and diminished oxidative respiration in vitro. In addition, the altered iron distribution increases sensitivity to ferroptosis, an iron-dependent mechanism of cell death, which can be reversed by restoring BDH2 expression or by treatment with the siderophore 2,5-DHBA [207].

## Nucleus-mitochondria contact sites

**Fig. 4:**
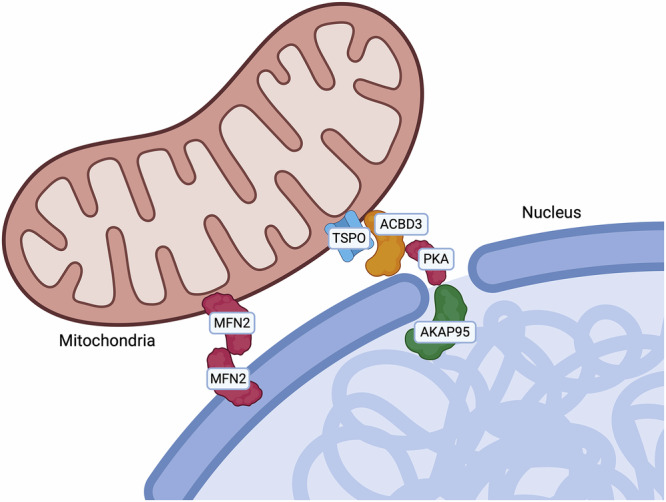
Nucleus-mitochondria. During stress response, mitochondria relocate around the nucleus and create stable contact sites with the nuclear membrane. TSPO, overexpressed in many tumours, tethers mitochondria to the nucleus to deliver cholesterol, sustaining NF-κB activity and promoting cell survival. MFN2 mediates mitochondria repositioning near the nucleus in response to growth signals.

Mitochondrial retrograde signalling enables mitochondria to communicate with the nucleus and orchestrate adaptive transcriptional programmes in response to cellular stress. This communication is facilitated by the formation of mitochondria–nucleus contacts (Fig. 4 and Table 4). Under stress conditions, mitochondria redistribute to the perinuclear region and form stable contacts with the nuclear envelope [210], promoting nuclear integrity and the expression of pro-survival genes, mainly *via* activation of NF-κB, modulation of cholesterol, production of ROS, and regulation of Ca^2+^ levels [211]. The first tether identified was the translocator protein TSPO, which is overexpressed in many tumours, where it binds cholesterol and it is involved in the suppression of mitophagy. This protein interacts with the A-kinase anchoring protein acyl–coenzyme A binding domain containing 3 (ACBD3) and the protein kinase A (PKA), which both complex with the nucleus *via* the A-kinase-anchoring protein (AKAP95). This tether facilitates cholesterol delivery to the nucleus, thereby sustaining NF-κB activity by preventing its deacetylation and reinforcing cell survival. In breast cancer cells, TSPO overexpression correlates with increased nucleus-mitochondria contacts and enhanced NF-κB recruitment [210, 211]. Additionally, MFN2 has been implicated in nucleus-mitochondria contacts. Proliferative signals induce the redistribution of mitochondria around the nucleus and concomitantly they trigger an MFN2 enrichment at mitochondria–nucleus interface [212]. This observation supports a model in which MFN2 mediates mitochondrial repositioning towards the nucleus to facilitate growth stimuli. In vitro, upon proliferative signals, nucleus-mitochondria contacts also permit translocation of the pyruvate dehydrogenase complex (PDC) into the nucleus, where it interacts directly with Lamin A [213].

**Table 4:** Main nucleus-mitochondria contact sites and their associated features.

Cargo	Main tether (nucleus-mito)	Biological functions	Tumour phenotype	Ref.
**Cholesterol**	TSPO-ACBD3	Transport of cholesterol to the nucleus	Mitophagy inhibition, increase of nucleus-mitochondria contacts, enhanced Nfkb recruitment	[210, 211]
	MFN2	Mitochondrial repositioning towards the nucleus		[231]

## ER-Peroxisomes contact sites

**Fig. 5:**
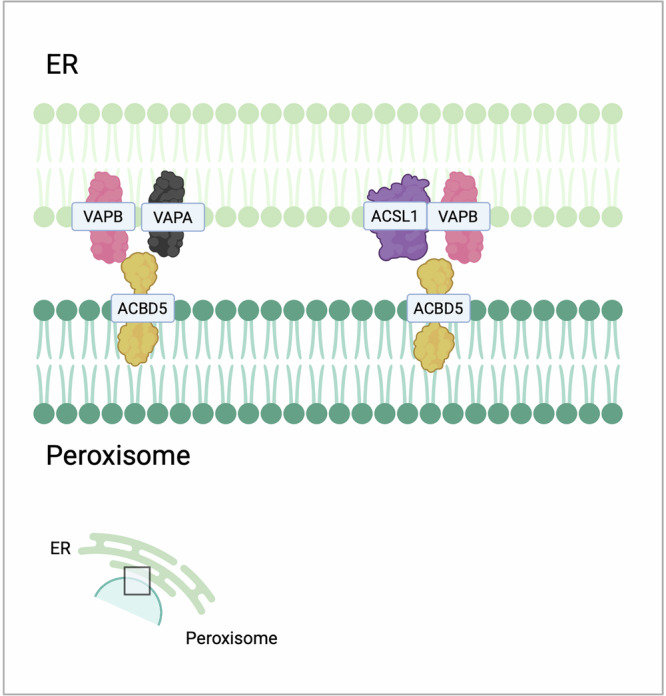
Peroxisomes-endoplasmic reticulum. Peroxisomes are critical organelles for lipid metabolism and interact closely with the ER. Key tethering proteins include ACBD5, which binds to VAPs to facilitate lipid transfer and peroxisome expansion. ACSL1, another crucial protein, forms contacts between the ER and peroxisomes and supports fatty acid β-oxidation and branched-chain fatty acid metabolism. In cancer, dysregulation of ACSL1 impacts proliferation, migration, EMT, and energy metabolism through AMPK activation.

Peroxisomes are essential organelles that play critical roles in lipid and hydrogen peroxide metabolism [214]. The interactions between peroxisomes and the ER are vital for lipid metabolism, with peroxisomes relying on the ER for their lipid composition [214] (Fig. 5 and Table 5). Approximately 70% of peroxisomes in mammalian cells have been observed to associate with the ER, facilitating the direct lipid transfer between these organelles [215], the ether-phospholipid biosynthesis (a process initiated in peroxisomes and completed in the ER), and the synthesis of polyunsaturated fatty acids (PUFAs), essential for membrane plasticity modulation and peroxisome dynamics [216]. Additionally, peroxisomes participate in cholesterol trafficking to the PM, with the ER potentially serving as an intermediate organelle [217]. Since cholesterol alteration is reported to contribute to cancer development, this axis may have a role in influencing tumourigenesis.

**Table 5:** Main ER-peroxisome contact sites and their associated features.

Cargo	Main tether (ER-perox)	Biological functions	Tumour phenotype	Citations
**Lipids**	ACBD5-VAPs	Lipid transfer, metabolic cooperation between peroxisomes and ER, FAO and phospholipid biosynthesis, peroxisome movement and positioning		[215, 218, 219]
**Fatty acids**	ACSL1	Contact formation, branched-chain fatty acid metabolism	Increase in cancer cell proliferation and migration, EMT promotion	[220, 221]

Recent studies elucidated the mechanisms underlying these interactions, emphasising the significance of acyl-coenzyme A-binding domain protein 5 (ACBD5) as a key tethering protein. ACBD proteins facilitate the establishment of contacts through their interaction with VAPs, thereby enhancing lipid transfer and metabolic cooperation between peroxisomes and the ER, essential for maintaining cellular metabolism, including FAO and phospholipid biosynthesis [218, 219]. The activity of this axis regulates peroxisome movement and positioning, suggesting that contacts can influence organelle motility and, consequently, cellular homeostasis [215]. Moreover, the VAPA-ACBD5 axis appears to be necessary for peroxisome growth since it exploits lipid transfer for membrane expansion. Interestingly, overexpression of ACBD5 has been associated with peroxisomal elongation in a VAP-dependent manner in vitro [217].

Long-chain acyl-CoA synthetase 1 (ACSL1) facilitates the transfer of fatty acids into the mitochondria for subsequent β-oxidation [220]. In the ER, ACSL1 establishes interactions with several peroxisomal proteins, such as ACSL1, VAPB, and ACBD5, promoting the formation of contacts. Furthermore, ER-targeted ACSL1 appears to interact with proteins involved in branched-chain fatty acid metabolism [220]. Various tumours are characterized by impaired expression of ACSL1. In colorectal and endometrial cancer cells, the absence of ACSL1 negatively impacts cancer cell proliferation and migration, while its upregulation correlates with EMT both in vitro and in vivo [221]. Additionally, ACSL1 may promote cancer cell proliferation and metastatization through the activation of AMP-activated protein kinase (AMPK) [222], that in turn can induce FAO, resulting in ATP production and impaired normal physiological processes in cancer cells [223].

## Lipid droplets contact sites

Lipid droplets (LDs) are spherical dynamic organelles which can be found in different cell types [224]. They are composed by a core of neutral lipids surrounded by a monolayer of phospholipids [225] and proteins belonging to the perilipin (PLIN) family which participate both in the synthesis and hydrolysis of the neutral lipids of the LD core [224]. Based on their localisation, LDs can be classified in cytoplasmatic, nuclear or luminal [226]. In mammals, LDs can be found free in the cytosol, or they can keep a functional connection with other organelles [227]. Membrane contacts between ER and LDs have been described to support lipid transport and homeostasis (Table 6). In detail, proteins of the ORPs family have been proven to participate in these ER-LD contacts [133]. ORP2 localises on the surface of LDs and it can bind VAPs, thus regulating LDs neutral lipid turnover and LDs positioning [228]. Furthermore, ORP5 localises at ER-LDs contacts where it mediates the counter-transport of PS and PI(4)P between ER and LDs, finally impacting on LDs growth [229]. Interestingly, ORP5 and ORP8 located at MERCs take part in the local lipid droplet biogenesis and maintenance, becoming key players at the triple interface among ER, mitochondria and LDs [230]. Moreover, these triple connections, which seemed to facilitate lipid anabolic pathways rather than FAO, correspond to a peculiar MERC proteome with an enrichment in proteins involved in lipid metabolism, including long-chain acyl-CoA metabolism, and metabolite processing from pyruvate [231].

**Table 6:** Main lipid droplets contact sites and their associated features.

Cargo	Main tether (LDs)	Biological functions	Tumour phenotype	Ref.
	CPT1A-PLIN2	Lipid metabolism		[225]
	PLIN5-FATP4	Binding of fatty acids, interaction with mitochondria, fatty acid trafficking, acyl-CoA synthesis	Increased FAO	[233]
	MIGA	Regulation of de novo synthesis of triglycerides		[67]
	VPS13D-TSG101	Trafficking of fatty acids from LDs to mitochondria		[236]

**Fig. 6:**
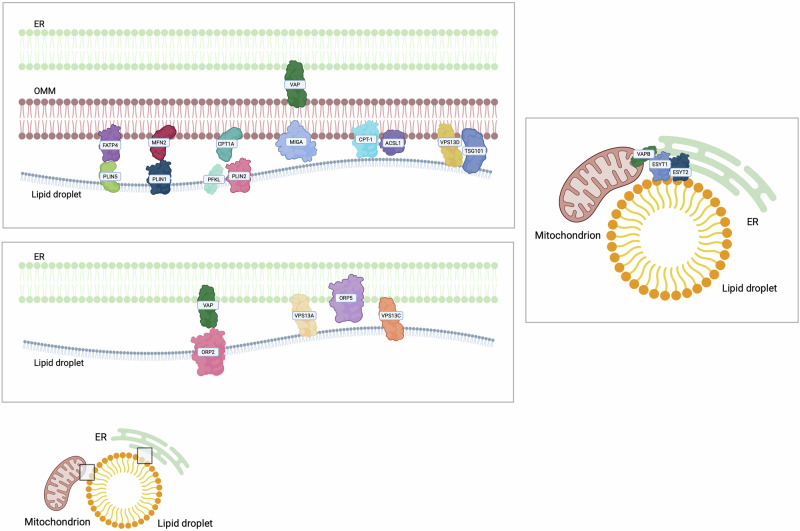
Lipid droplets. Lipid droplets (LDs) are spherical organelles with a neutral lipid core and a phospholipid monolayer, coated by PLIN proteins that regulate lipid storage and mobilisation. LDs interact with the ER through ORP2, ORP5, and VAPs to control lipid transfer. They also contact mitochondria *via* proteins like CPT1A, PLIN2, FATP4, and ACSL1 to support fatty acid oxidation, especially under stress. Multi-organelle contacts involving MFN2, MIGA, ESYT1/2, and VPS13 proteins coordinate lipid metabolism. Since LDs are abundant in cancer cells, their contact networks may serve as potential targets for therapy.

Apart from the ER, LDs can bind other organelles, and they have been proven to modulate many cellular processes (Fig. 6). LDs can form contacts with mitochondria, whose increase during high levels of energy demand [232]. One of these contacts involves OMM Carnitine palmitoyltransferase 1 (CPT1A) that binds PLIN2 on the LDs. In vivo, this interaction becomes stronger upon glucose starvation and involves PFKL, an LD-associated protein which is highly expressed in HCC [225]. In hepatic tissues, a key player in the transfer of fatty acid for oxidation from LDs to mitochondria is ACSL1 which can interact with CPT1A. This interaction involves also synaptosomal-associated protein 23 (SNAP23) [219]. Similarly, Perilipin 5 (PLIN5) is a LD protein which could bind fatty acids, and is involved in the interaction with mitochondria, by interacting with the OMM Long Chain Fatty Acid Transporter Protein 4 (FATP4), finally supporting fatty acid trafficking. Interestingly, FATP4 participates both in acyl-CoA synthesis and in fatty acid transport from LDs to mitochondria during starvation, thus promoting FAO in sarcoma cells [233]. In addition, PLIN5 can interact with MFN2 [233], which promotes the crosstalk between LDs and mitochondria in brown adipocytes [234].

Finally, VSP13A and VSP13C localise at the ER-LDs interface [235], while VPS13D localises at LDs-mitochondria in response to oleic acid after starvation. In liver cancer cells, VPS13D downregulation causes a decrease in the degree of association between mitochondria and LDs, thus highlighting a role for VPS13D in membrane tethering [236]. Moreover, VPS13D is involved in the trafficking of fatty acids from LDs to mitochondria through the interaction with Tumour Susceptibility Gene 101 (TSG101) [236]. Interestingly, an interaction between LDs and the Trans Golgi Network (TGN) has been demonstrated to be mediated by VPS13B [237]. Importantly, since cancer cells display many LDs, which represent a source of energy and lipid precursors for rapid cancer cell proliferation and for the synthesis of membranous organelles, the LD contactome would become very important as possible oncological target [224].

## ER-Plasma membrane contact sites

**Fig. 7:**
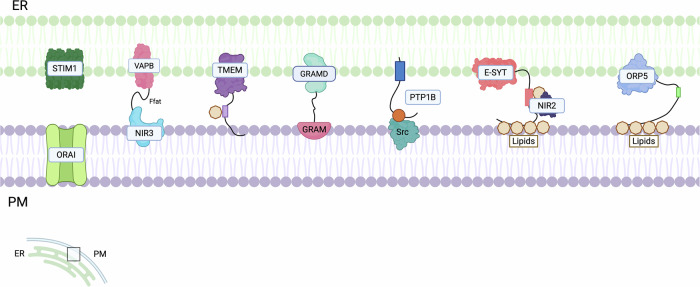
Endoplasmic reticulum-plasma membrane. ER-PM contact sites are essential for the control of calcium signalling and lipid exchange, involving key proteins like STIM1, ORAI channels, and lipid transfer proteins. STIM1-driven calcium entry influences cancer by enhancing cell proliferation, migration, and blood vessel formation. Other proteins at these junctions, including PTP1B, E-Syt, and Nir2, affect tumour growth and spread. Additionally, anoctamin (TMEM16 family) and GRAMD proteins contribute to cancer progression by regulating lipid balance and cellular communication.

In addition to mitochondria, lysosomes and LDs, the ER can interact with the PM. Initial detection of ER-PM contacts occurred in muscle cells in the mid-20^th^ century [238], and they comprise small focal points and larger cisterns, facilitating interactions between protein and lipid components of both membranes [239]. A variety of tethering proteins are in these proximity regions, and they exert crucial functions for cellular microenvironment maintenance [86] (Fig. 7 and Table 7). ER-PM contacts play a role in the modulation of non-vesicular Ca^2+^ and membrane lipid transport, particularly during depletion of ER or cytosolic Ca^2+^ levels. STIM proteins are integral to the SOCE and subsequent Ca^2+^ signalling [240]. In vertebrates, two human STIM proteins can be found: STIM1 and STIM2. Both proteins are expressed in various cell types and serve as ER Ca^2+^ sensors; however, STIM2, unlike STIM1, is exclusively localised to the ER and appears to be a weaker activator of OraI1. STIM1 is normally inhibited, but it is activated upon Ca^2+^ depletion, while STIM2 is characterised by its heightened sensitivity to minor fluctuations in ER Ca^2+^ concentration [241]. STIM1 acts as a tether between the ER and the OraI channels on the PM when ER Ca^2+^ concentrations decline [242]. Upon Ca^2+^ depletion, it undergoes oligomerization and activates OraI1 at the PM, promoting Ca^2+^ influx to replenish the ER [243]. Moreover, STIM1 interacts with adenylate cyclase, an essential signalling molecule downstream of G protein-coupled receptors, leading to the generation of cyclic AMP (cAMP) in a process named storage-operational cAMP signalling (SOcAMPS). The interplay between Ca^2+^ and cAMP signals at ER-PM contacts is critical for cell homeostasis [244]. SOCE derived from the STIM1–Orai1 axis plays a critical role in multiple aspects of tumour biology and has been implicated in cancer progression [245]. Elevated STIM1 expression correlates with increased metastatic potential and reduced overall survival, and although the mechanisms remain incompletely defined, STIM1 appears to regulate cell migration and proliferation. Silencing STIM1 in cervical cancer cells induces cell cycle arrest and suppresses proliferation [246]. Particularly, STIM1-mediated SOCE enhances cell migration and metastatic behaviour, contributing to breast cancer progression [247], while in vivo studies indicate that STIM1 overexpression promotes angiogenesis and VEGF production in cervical cancer [246]. Consistent with these pro-tumoural functions, STIM1/Ca²⁺ signalling inhibits apoptosis in pancreatic cancer, gastric cancer, TNBC, and HNSCC. In pancreatic cancer cells, downregulation of OraI1 or STIM1 increases the sensitivity to chemotherapeutic agents [248]. Similarly, in breast cancer cell lines, the knockdown or the inhibition of the STIM1–Orai1 axis limits proliferation and metastatic potential [249]. These findings align with observations in HNSCC, where STIM1 downregulation induces apoptosis and suppresses proliferation [250]. In contrast, in prostate cancer, the STIM1–Orai1 axis exhibits an opposing role, as OraI1 downregulation appears to protect cells from apoptosis in vitro, despite regulating cell proliferation and migration [251]. OraI3 is also dysregulated in malignancies, being overexpressed in breast and prostate cancer if compared to normal tissues [252], and OraI3-dependent SOCE is required for specific phases of the cell cycle in oestrogen-expressing cells [253]. Additionally, lipid transfer between the ER and PM mediated by lipid transfer proteins (LTPs) and vesicular trafficking, is essential for maintaining distinct lipid compositions. Specific proteins, such as ORPs, are responsible for transferring cholesterol and PS from the ER to the PM [254], and several members of the family have been related to cancer [228, 255–258]. Furthermore, there are proteins critical for preserving PM integrity, including Scs2/22 (VAP orthologs), E-Syt proteins, and Ist2. Protein tyrosine phosphatase 1B (PTP1B) is anchored to the ER and catalyses the dephosphorylation of various substrates at ER-PM contacts, thereby influencing cellular adhesion [259]. PTP1B also affects tumour growth, metabolism, and metastatic potential through interactions with different substrates, which can positively or negatively influence tumour development [260]. Among the interacting partners of PTP1B are AKT and the tyrosine-phosphorylated insulin receptor, suggesting that PTP1B may play a role in insulin signalling pathways and in glucose metabolism regulation [261]. PTP1B exhibits context-dependent roles in cancer, acting either as a tumour promoter or suppressor depending on the tissue type and signalling environment. In several cancers, including gastric, prostate, NSCLC, hepatocellular, and colorectal tumours, PTP1B is frequently overexpressed, and this overexpression is associated with increased metastatic potential and reduced patient survival [262]. PTP1B can enhance tumour progression by binding to Src and activating its kinase activity, thereby promoting cell migration and metastatic dissemination in vitro [220], as well as by stimulating tumour cell proliferation, as shown in colon cancer cells [221]. Conversely, evidence also suggests tumour suppressive functions for PTP1B in distinct cancer types. These effects are most clearly characterised in melanoma and glioblastoma, where PTP1B contributes to caveolin dephosphorylation and reduces cell migration in vitro [263]. In addition, loss or reduction of PTP1B expression favours tumour development in ovarian cancer and B-cell lymphoma in vitro, where the PTP1B antagonises BRK and IGF-1R signalling [264]. The context-dependent nature of PTP1B function is further highlighted by observations that low PTP1B mRNA levels associate with poor cellular differentiation in some tumour types, whereas the opposite correlation occurs in others. Collectively, these findings indicate that the role of PTP1B in cancer is highly tissue-specific and dependent on the surrounding signalling context [222].

**Table 7:** Main ER-plasma membrane contact sites and their associated features.

Cargo	Main tether (ER-PM)	Biological functions	Tumour phenotype	Ref.
**Ca**^**2+**^	STIM1–Ora1 STIM2–Ora1	ER Ca^2+^ signalling, SOCE pathway, cell homeostasis	Increased metastatic potential, reduced overall survival, promotion of cell migration and proliferation, apoptosis modulation	[240–244, 246, 331]
**Cholesterol**	ORPs	Cholesterol and phosphoserine transfer from ER to PM		[254]
	PTP1B	Cellular adhesion, insulin signalling pathway, glucose metabolism regulation	Tumour growth enhancement or reduction depending on the tumour type, metastatic potential increase	[260, 262, 264, 332–334]
**Diacylglycerol**	E-Syt proteins	Lipid transfer, SOCE pathway	Impact on tumourigenic potential	[265–269]
	TMEM16A	Receptor signalling regulation	Increased cell proliferation	[270–276]
	Nir2 and Nir3 - VAPs	Phospholipid homeostasis, modulation of PI3K/Akt signalling pathway	EMT promotion, metastasis formation	[277–280]
**Cholesterol**	GRAMD1	Lipid transfer, sterol homeostasis	Impact on mitochondrial bioenergetics and positive contribute to cancer progression when localised in MAMs	[60, 282]
**Ca2+**	GRAMD2	Ca^2+^ transfer, SOCE pathway	Impact on mitochondrial bioenergetics and positive contribute to cancer progression when localised in MAMs	[60, 281]

E-Syt proteins are located at ER-PM contacts and facilitate the transfer of phospholipase C (PLC)-generated diacylglycerol from the PM to the ER [265]. In mammalian cells three different isoforms, termed E-Syt1, E-Syt2, and E-Syt3, are expressed: E-Syt2 and E-Syt3 can form ER-PM contact sites in a Ca^2+^-independent manner, while E-Syt1 requires elevated Ca^2+^ levels [266]. In vivo experiments show that when Ca^2+^ is transferred through the SOCE pathway or voltage-gated Ca^2+^ channels, E-Syt1 is recruited to ER-PM contacts. Additionally, Ca^2+^ induces a reduction in the length of ER-PM contact sites at E-Syt1-dependent junctions [267, 268]. This finding underscores the role of these proteins in response to external stimuli and highlights the significance of the crosstalk between the ER and PM [86]. The role of E-Syt proteins has also been investigated in cancer, where E-Syt1 induces the secretion of protein kinase C delta (PKCδ). This occurs through growth signals, including insulin-like growth factor 1 receptor (IGF1R) and extracellular signal-regulated kinase 1/2 (ERK1/2) and these stimuli directly influence the tumourigenic potential. Notably, phosphorylation of IGF1R and ERK1/2 was found to decrease in E-Syt1-deficient cells compared to control cells during nutrient starvation [269].

Another ER-PM contact is mediated by Anoctamin, TMEM16 and Ist2p homologues, membrane proteins that dimerise within the membranes of ER and PM. The cavity formed between the subunits contains lipid molecules, which are involved in the lipid transfer process from one bilayer to another. Many of these proteins, such as TMEM16a/b, localise to the PM and function as Ca^2+^-activated Cl^−^ ion channels. Other homologues, including TMEM16f and TMEM16, appear to possess phospholipid scramblase activities [270, 271]. Several studies report that anoctamin proteins are highly expressed in different tumours. Specifically, TMEM16aA seems to be overexpressed in gastrointestinal stromal tumours [272] and oral squamous carcinoma [273]. In some cancers, its expression correlates with cell proliferation rate even if the results are contradictory and they depend on the cell type [274]. Even though the relationship between TMEM16a expression and neoplastic cell growth remains poorly understood, it has been proposed that it may interfere with signalling pathways involved in cancer progression. For example, in gastrointestinal stromal tumour cells, reduced TMEM16a expression led to the upregulation of insulin-like growth factor-binding protein 5, an antiangiogenic factor [275]. In breast cancer, TMEM16a expression appears to correlate with epidermal growth factor receptor signalling and calmodulin-dependent kinase activity [276].

In addition, the phosphatidylinositol-transfer protein Nir2 and Nir3 play critical roles in phospholipid homeostasis at ER-PM junctions [86], by binding of the VAPs [277]. Regarding the role of Nir2 in cancer, it has been observed that it potentiates EMT in mammary cells, so it is required for the formation of lung metastasis in breast cancer [278], by influencing the PI3K/AKT and ERK signalling pathways, which contribute to breast cancer development and progression [279, 280]. Recently, Nir2 has been shown to alter cell proliferation through its interaction with VAPB, which regulates breast tumour cell proliferation and AKT activation both in vitro and in vivo [60].

Finally, GRAMD protein isoforms like GRAMD1 in mammals contain StART-like lipid transfer domains, while GRAMD2 and GRAMD3 do not participate in lipid transfer [60]. These proteins are anchored to the ER and target the PM [281]. GRAMD1 proteins play a critical role in PM sterol homeostasis, as they can interact with free cholesterol and facilitate its transport to the ER [282]. Conversely, GRAMD2 appears to be involved in Ca^2+^ homeostasis and the SOCE pathway through the recruitment of STIM1 [281]. In cancer, it has been proposed that GRAMD proteins may influence mitochondrial bioenergetics and cancer progression by regulating cholesterol levels and facilitating the transport of cholesterol between mitochondria and ER [283]. Notably, the absence of GRAMD1C has been associated with increased autophagic flux, elevated cholesterol levels in mitochondria, as well as enhanced mitochondrial respiration, finally contributing to cancer progression [283].

## Contact sites and immune regulation

The regulation of membrane contact sites has emerged as a fundamental factor of immune responses in cancer. A key example is the role of mitochondria–ER contacts in modulating the efficacy of cytotoxic anticancer therapies. In parallel, the organisation of the cancer cell glycocalyx critically shapes the capability of cytotoxic lymphocytes to engage and eliminate malignant cells. One study demonstrated that glioma stem-like cells with reduced surface glycan presence are markedly more susceptible to lymphocyte-mediated cytotoxicity. Notably, these cells also display diminished mitochondria–ER contacts, leading to decreased mitochondrial Ca²⁺ uptake and defective lipid biosynthetic pathways required for maintaining proper glycolipid composition at the cell surface [63, 284–287]. Disruption of ER-endosome contact sites has also emerged as a pivotal trigger of NLRP3 inflammasome activation, as their disruption drives endosomal PI4P accumulation and impairs endosome-to-Golgi trafficking. These events facilitate NLRP3 recruitment to endosome, revealing membrane contact sites integrity as a key regulator of innate immune activation both in vitro and in vivo [288]. Moreover, ER stress has been shown to strengthen ER–mitochondria contacts, thereby promoting Ca²⁺-dependent NLRP3 activation in peripheral monocytes and central nervous system microglia [289].

Collectively, these findings highlight a complex and dynamic network of organelle contact sites that orchestrate immune signalling pathways in cancer and inflammatory contexts.

## Multiorganellar junctions

The intracellular contact sites described above can co-exist and dynamically assemble to generate triple and quadruple interconnections. The functional organisation of such specialised hubs coordinates the cellular response to different metabolic or signalling stimuli [285, 290]. Among these, innate immune pathways are prominently regulated, with membrane contact sites playing a central role in paraptosis‑mediated immunogenic cell death [291, 292], in cGAS– Stimulator of Interferon Gene (STING) axis, a key sensor of aberrant cytosolic DNA [284], and the unfolded protein response (UPR) triggered by ER stress [10]. Indeed, the most characterised triple interconnections involve ER, mitochondria and lysosomes/endosomes [64, 235, 293], ER, mitochondria and PM [294] and ER, mitochondria and LDs [67, 231, 232, 295].

In particular, the Protrudin interactor PDZD8 is an ER transmembrane protein that interacts with Rab7 and Protrudin at ER–late endosome contacts [293]. Notably, PDZD8 has also been identified at MERCs [296]. Emerging evidence suggests that PDZD8 may act as a shared component between these two distinct membranes contact sites, contributing to the formation of three-way contacts between the ER, mitochondria, and late endosomes [293]. PDZD8 is upregulated in stomach cancer tissue compared with normal tissue, contributing to cancer cells proliferation and metastasis and has therefore been proposed as a potential therapeutic target [297].

In adipocytes, LDs are involved in three-way contacts with mitochondria and the ER. Indeed, MIGA, an OMM protein, interacts with LDs and with VAPA/B on the ER, thus regulating the de novo synthesis of triglycerides in vitro [67]. Another triple membrane contact between ER, mitochondria and LDs is constituted by a multimeric protein complex composed by ESYT1, ESYT2 and VAPB [232]. This contact site facilitates the transfer of fatty acids to mitochondria for β-oxidation, as observed in liver cancer cells. Disruption of this complex impairs the utilisation of lipid droplet-derived fatty acids, remodels cellular lipid metabolism, and induces lipotoxic stress [232].

MERCs have also been shown to establish contacts with the PM. Specifically, the ER-mitochondria-PM triple interaction has been found to regulate EGFR signalling. Non-clathrin mediated endocytosis of EGFR, activated at high ligand concentrations and essential for receptor degradation, is regulated by ER-derived Ca^2+^ oscillations upon receptor engagement. These localised Ca^2+^ microdomains increase mitochondria metabolism and ATP production, which drive cortical actin remodelling necessary for endocytic vesicle formation [294].

Interestingly, multi-organellar contact sites can reshape both the proteome and the functionality of the involved organelles, *e.g.* in the case of MERCs interacting with LDs [231] or peroxisomes contacting the MAMs which are enriched in pyruvate dehydrogenase (PDH) [298, 299]. Moreover, the synthesis of inflammatory lipid mediator has been associated with fatty acid shuttling among the mitochondria, ER, peroxisome and LDs [287].

## Chemical modulation of organelle contact sites

Given the importance of several MCSs in modulating many signalling pathways also in cancer cells, it is not surprising that they are arising as possible oncological targets and different compounds able to modulate these contacts have been studied to possibly impair disease development. Indeed, MCSs may be modulated by several chemical compounds that can either influence the interaction between tethering proteins or inhibit their enzymatic activity. These compounds can directly interact with tethers or modulate their expression levels, as well as impact on signal transduction cascades that, in turn, can control the formation of MCSs. Some drugs have been shown to directly modulate membrane contact sites (Table 8), whereas other target proteins are implicated in contact site formation and consequently affects organelle function, although their direct role in contact site modulation has yet to be defined (Table 9).

**Table 8:** Drugs with a confirmed role in contact site modulation.

Drug	Target	Tether/Contact site	Biological outcome	Tumour	Ref.
**LDC-3/Dynarrestin**	PTPIP51	PTPIP51-VAPB Mito-ER	Stabilised mito-ER association. Reduced mitochondrial metabolic rate.	Breast cancer	[335]
**Resveratrol**	VDAC1, SERCA	Mito-ER	Induced mitochondrial Ca^2+^ overload and apoptotic cell death.	Cervical cancer	[303]
**ABT737**	Bcl-2 protein family	Mito-ER	Increased mito–ER contacts and induction of Ca²⁺ transfer–mediated apoptosis.	Ovarian cancer	[336]
**Metformin**	VDAC1	IP3R-GRP75-VDAC1 Mito-ER	Disrupted mito–ER interactions, reduced mitochondrial Ca²⁺ influx and ATP production, and induced autophagy-related tumour cell death.	Breast cancer and hepatocellular carcinoma	[337]
**Cisplatin**		Mito-ER	Increased mito–ER contact sites and induced Ca²⁺ transfer, causing mitochondrial calcium overload and triggering apoptosis.	Ovarian cancer	[338]
**Azaphenothiazine Derivatives**	GRP75	IP3R-GRP75-VDAC Mito-ER	Disrupted mito–ER interactions and reduced mitochondrial Ca²⁺ flux, decreased ATP production, and induced apoptosis	Endometrial cancer	[339]
**MKT-077**	GRP75	IP3R-GRP75-VDAC Mito-ER	Reduced mito–ER contact site formation and disrupted mitochondrial membrane potential and induced cell-cycle arrest and apoptosis.	Glioblastoma	[306]

**Table 9:** Drugs with indirect effects on organelles.

Drug	Target	Biological outcome	Tumour	Ref.
**Fluoxetine**	VDAC	Induced mitochondrial Ca^2+^ overload and cell death.	Hepatoma, ovarian adenocarcinoma, lung cancer, colorectal adenocarcinoma, neuroblastoma, medulloblastoma, breast carcinoma	[340]
**Aspirin**	VDAC	Altered Ca²⁺ homeostasis, dissipated mitochondrial membrane potential and promoted cell death.	Ovarian cancer, human endocervical epithelial cancer	[341]
**Erastin**	VDAC	Induced mitochondrial dysfunction and ROS release, leading to oxidative cell death.	Mutated tumourigenic fibroblasts	[342]
**Salinomycin**	TRPML1	Disrupted lysosomal iron homoeostasis and promoted ferroptosis.	Ovarian cancer	[193]
**Mdivi-1**	Drp1	Altered mitochondrial morphology and cellular oxygen consumption, increased mitochondrial membrane permeability and induced apoptosis.	Hepatocarcinoma	[202]
**Ned-19**	NAADP	Inhibited Ca²⁺ signalling and suppressed tumour growth and angiogenesis.	Melanoma	[312]
**OSW-1**	OSBP	Promoted endoplasmic reticulum stress and activated necroptotic cell death.	Colorectal cancer	[343]
**VS1**	STARD3	Antiproliferative activity.	Breast and colon cancer	[307]
**Cationic amphiphilic drugs (CADs)**		Induced lysosome-dependent cell death.	Breast cancer	[308]
**Bafilomycin**	V-ATPase	Inhibited lysosomal acidification and fusion. Blocked autophagy and induced apoptosis.	Lymphoblastic leukaemia	[344, 345]

Many identified pharmacological agents act on VDAC1, a component of the IP3R/GRP75/VDAC tethering complex located at the MERCs, effectively blocking its interactions. Other chemicals impact on VDAC activity by inhibiting the interaction between the channel and various interactors, such as hexokinases [300]. Some of these compounds, through their action on VDAC, exert a pro-apoptotic effect, as they depolarise mitochondria or induce cytochrome c release, thereby promoting apoptosis in leukaemia cells [301]. Conversely, certain compounds function as transcriptional modulators. The activity of these chemicals primarily affects proteins that reside at MERCs by facilitating RNA synthesis of MERC components [302].

Studies indicate that after the treatment with various compounds, including polyphenols, cancer cells exhibit an increase in the formation of MERCs and in Ca^2+^ transfer, which consequently triggers apoptosis [303]. Furthermore, certain chemical molecules impacting on various signalling pathways can modulate the structure of MERCs. It has been demonstrated that ER-stress enhances the interaction between the ER and mitochondria [304]. In the same way, other metabolic modifications can modulate the length of MERCs [304]. Moreover, there are molecules that can impact AMPK function, thereby modulating the activity or expression levels of proteins involved in MERCs [305]. In conclusion, several compounds that modulate the structure, stability, and composition of MERCs have been developed with the objective of influencing MERCs and impacting on pathological phenotypes.

Among the pharmacological agents known to influence the formation of MERCs, there is MKT-077, which exerts its effects by targeting GRP75, thereby modulating the IP3R-GRP75-VDAC axis, which subsequently impacts the Ca^2+^ flux from the ER to the mitochondria. Treatment with this agent in glioblastoma cells results in a significant impairment of mitochondrial membrane potential [306]. Such findings underscore the crucial role of MERCs in cellular physiology and highlight the potential therapeutic implications of targeting these interactions in the context of glioblastoma treatment.

Other chemicals can affect tethering players, including ER-lysosome ones. For instance, VS1, a STARD3 inhibitor, has been developed utilising an in silico approach. Experiments conducted to evaluate its effect on breast and colon cancer cell lines demonstrated that it has an antiproliferative effect [307]. Some drugs do not directly target tethers, but they may influence lysosomal functionality, the autophagic process, and the induction of cell death. For example, cationic amphiphilic drugs (CADs) can inhibit lysosomal enzymes, particularly lipases, thereby compromising the integrity of the lysosomal membrane in MCF7, A549, and HeLa cells [308]. Similarly, bafilomycin has been developed as a v-ATPase inhibitor, which inhibits lysosomal acidification and fusion. Consequently, these drugs can modulate lysosomal function and may indirectly affect ER-lysosome interactions [309].

At present, there are no compounds that specifically target the components of the axis between mitochondria and peroxisomes. However, such molecules may influence these contacts through the modulation of essential processes, such as the maintenance of mitochondrial membrane integrity, lipid metabolism, and peroxisomal function.

Finally, Ca^2+^ is an important messenger which activates different signalling pathways and cellular processes [310]. Kilpatrick et al. demonstrated how a tight regulation of Ca^2+^ fluxes can affect the formation of ER-endosomes contacts and can impact the morphology of late-endocytic pathway compartments in vitro [311]. In particular, Ned-19 emerged from a large-scale screening as the most powerful permeant NAADP inhibitor which abrogates NAADP-mediated Ca^2+^ release at 100 μM concentration without affecting Ca^2+^ release mediated by other molecules, thus highlighting its high selectivity. A Cconcentration–response curve suggests that Ned-19 acts as a functionally irreversible noncompetitive antagonist. The administration of this compound has been described to impact on melanoma growth, metastatic potential and angiogenesis in mice [312]. Similar results on the Ca^2+^-dependent endo-lysosomal ultrastructure modification have been obtained upon 2 hours of treatment with Ned-K, a Ned-19 analogue in which the fluoride has been substituted with a cyano group [313].

## Conclusions

**Fig. 8:**
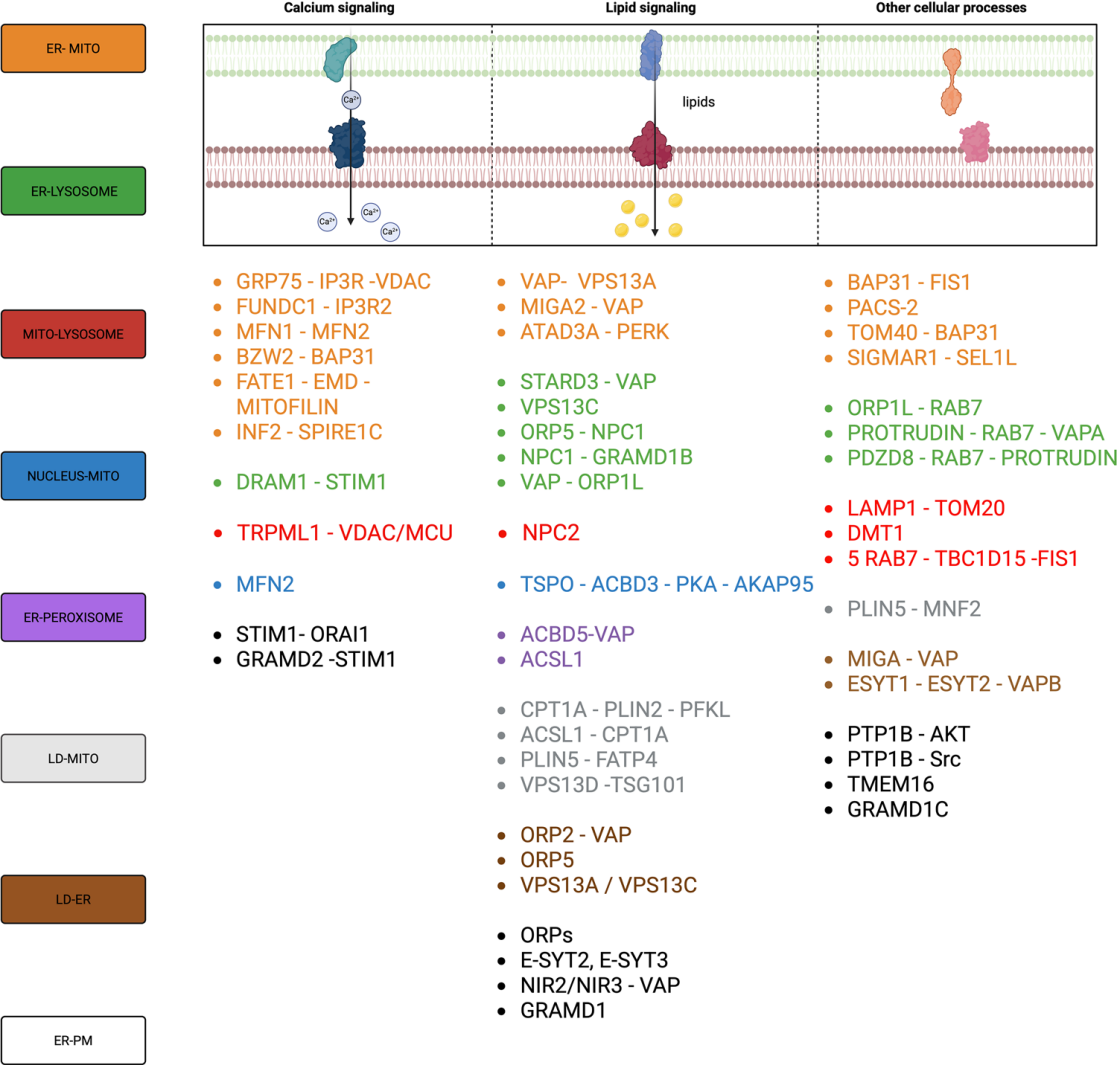
MCSs influence several key cellular processes. MCS facilitates inter-organelle communication and regulates essential processes, including intracellular signalling, metabolic coordination, molecular transport and, mainly, ion and lipid homeostasis. These processes and signalling pathways are fundamental to tumour development and aggressiveness. Mito-ER and ER–lysosome contacts represent the most extensively characterised tethers.

MCSs can be defined as the functional juxtaposition of cellular organelle membranes. These contacts are mediated by proteins that stabilise the interaction and are involved in the functions of the tethering axis. The most well-characterised interactions are MERCs and ER-lysosomes, while new interactions involving the ER, the PM and LDs, among others, are now emerging as new hubs for organelle communication. It has been proven that MCSs affect a plethora of cellular functions, including intracellular signalling, cellular metabolism, transport of Ca^2+^ and lipid homeostasis (Fig. 8). Therefore, they have been shown to play a role in various physiological and pathological processes. In cancer, modulation of MCSs affects tumour progression and the aggressiveness of cancer cells. However, this research field remains largely unexplored, and further studies are required to clarify the contribution of contacts to the progression of different types of cancer and their impact on cell death sensitivity and drug resistance. Furthermore, modulating contacts using different compounds could represent a promising new therapeutic strategy for overcoming these malignancies. However, few strategies have been reported, and not all these inter-organelle crosstalk hubs have been fully described in a tumour context, thus highlighting the necessity for further studies to shed light on these new, promising oncological targets.
